# Analysis of the milk kefir pan-metagenome reveals four community types, core species, and associated metabolic pathways

**DOI:** 10.1016/j.isci.2023.108004

**Published:** 2023-09-21

**Authors:** Liam H. Walsh, Mairéad Coakley, Aaron M. Walsh, Fiona Crispie, Paul W. O’Toole, Paul D. Cotter

**Affiliations:** 1 Teagasc Food Research Centre, Moorepark, Fermoy, Co. Cork, Ireland; 2 School of Microbiology, University College Cork, Ireland; 3 APC Microbiome Ireland SFI Research Centre, University College Cork, Ireland; 4 VistaMilk SFI Research Centre, Teagasc, Moorepark, Fermoy, Co. Cork, Ireland

**Keywords:** Geographic areas, Microbial genomics, Microbial metabolism, Genomics

## Abstract

A comprehensive metagenomics-based investigation of the microorganisms present within milk kefir communities from across the globe was carried out with a view to defining the milk kefir pan-metagenome, including details relating to core and non-core components. Milk kefir samples, generated by inoculating full fat, pasteurized cow’s milk with 64 kefir grains sourced from 25 different countries, were analyzed. We identified core features, including a consistent pattern of domination by representatives from the species *Lactobacillus helveticus* or the sub-species *Lactobacillus kefiranofaciens* subsp*. kefiranofaciens*, *Lactococcus lactis* subsp*. lactis* or *Lla. cremoris* subsp*. cremoris* in each kefir. Notably, even in kefirs where the lactococci did not dominate, they and 51 associated metabolic pathways were identified across all metagenomes. These insights can contribute to future efforts to create tailored kefir-based microbial communities for different applications and assist regulators and producers to ensure that kefir products have a microbial composition that reflects the artisanal beverage.

## Highlights

• Collectively 46 species microbial species were detected in the milk kefir microbiome
• Globally, milk kefir microbiomes cluster into four groups
• These groups have distinct, dominant taxa, and do not exhibit geographic signatures
• *Lactococcus lactis* and *Lactococcus cremoris* were core to the pan-metagenome

## Graphical abstract

**Figure:**
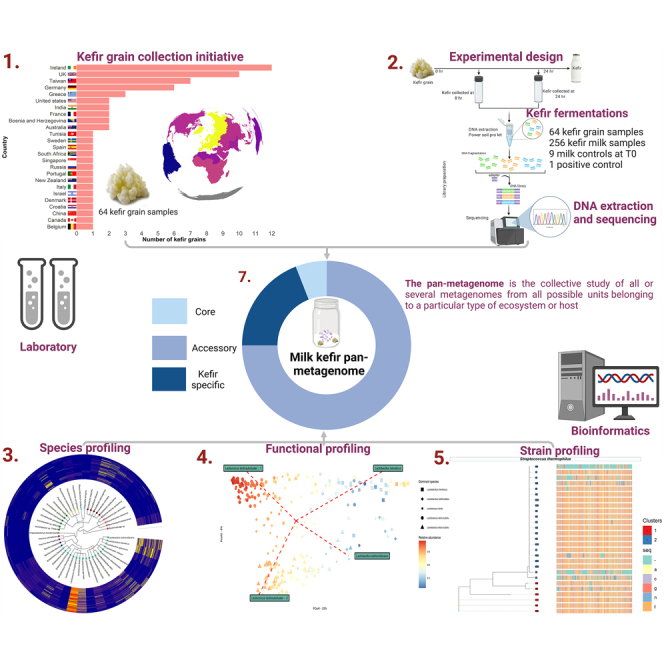

## Introduction

Milk kefir is a fermented milk product traditionally produced by inoculating milk with a kefir grain, which is a protein and exopolysaccharide matrix containing a variety of microorganisms, including those responsible for the fermentation process.^1^ The live cultures represent a mixture of microbial species, including, for example, representatives from the genera *Lactobacillus*, *Lactiplantibacillus*, *Lentilactobacillus*, *Lactococcus*, *Leuconostoc*, *Streptococcus*, *Kluyveromyces*, and *Saccharomyces*.^2^ Previous research efforts have verified the presence and dominance of kefiran producing bacteria, such as *Lactobacillus kefiranofaciens* and *Lentilactobacillus kefiri* within the milk kefir grain microbiome.^3^ This pattern of dominance has been verified through the microbial analysis of kefir grains from different geographic regions, with heterogeneity typically arising from low-abundance bacterial and eukaryotic communities.^4^^,^^5^ In contrast, the resulting fermented milk known as ‘milk kefir’ has been reported to have a more varied compositional profile compared to the grain^2^ and can undergo substantial compositional changes within a few hours.^5^^,^^6^ The organoleptic and health-promoting properties of kefir are attributed to the food’s nutritional content, the fermenting microorganisms and the by-products (including postbiotics) produced from their metabolic activities.^7^ Numerous studies examining the effects of milk kefir on host health have reported positive results.^2^^,^^8^ Indeed, milk kefir consumption has been reported to reduce the symptoms of lactose intolerance in humans,^9^ while testing in rodent models has indicated that specific milk kefirs and/or components thereof can contribute to wound healing, reduce cholesterol, impact the gut-brain axis and demonstrate anti-carcinogenic, anti-obesity, anti-inflammatory or anti-pathogenic properties. However, in many instances, the mechanisms for these purported effects have not been elucidated.^10^^,^^11^^,^^12^^,^^13^^,^^14^^,^^15^^,^^16^ Kefir-associated microbes can also be employed in fermented food production to improve sensory characteristics and/or enhance microbiological safety.^17^^,^^18^^,^^19^ To further optimize milk kefir, and the combination of microbes responsible for its production, it is necessary to first understand and harness the heterogeneous microbial communities present in different beverages, which can extend to different batches of the same product.^20^ This is necessary as it is also clear that at least some health benefits are not universal, but are associated with specific grains and, thus specific microbes,^14^ with the latter providing the justification for isolating specific strains and creating simple ‘pitched’ communities that retain the key traits of the beverage, while facilitating greater quality control.^21^ Another challenge relates to regulations, with inconsistencies across different jurisdictions regarding what constitutes a milk kefir and, in other cases, a lack of regulation,^22^ which can lead to products being marketed as kefir without having a microbial composition that reflects the artisanal beverage.^23^ Metagenomics-based identification and characterization of the microorganisms present within kefir communities of different origin has the potential to define the milk kefir pan-metagenome. According to Imchen et al.*,* a pan-metagenome is “the collective study of all or several metagenomes from all possible units belonging to a particular type of ecosystem or host”.^24^ Exploration of the pan-metagenome of milk kefir can provide considerable insights into the heterogeneity of milk kefir microbial populations, while also uncovering key core or conserved features. This information might also help to identify genes, and ultimately mechanisms, which contribute to health benefits. In milk kefir, sampling of both the milk kefir grain and liquid microbiome can provide insights into the pan-metagenome. However, the fermented beverage often represents a more varied compositional profile, which can include the residual milk microbes that persist in the fermentation medium, and as such represents the more informative kefir microbial community to study microbial diversity.^5^ Here we describe the metagenomics-based characterization of milk kefir generated by 64 kefir grains sourced from 25 different countries, providing a framework upon which the minimal microbial and functional criteria to define a milk product as a milk kefir can be established through the identification of a core pan-metagenome in milk kefir fermentations.

## Results

### Taxonomic profiling reveals distinct community types in the kefir microbiome

**Figure 1:**
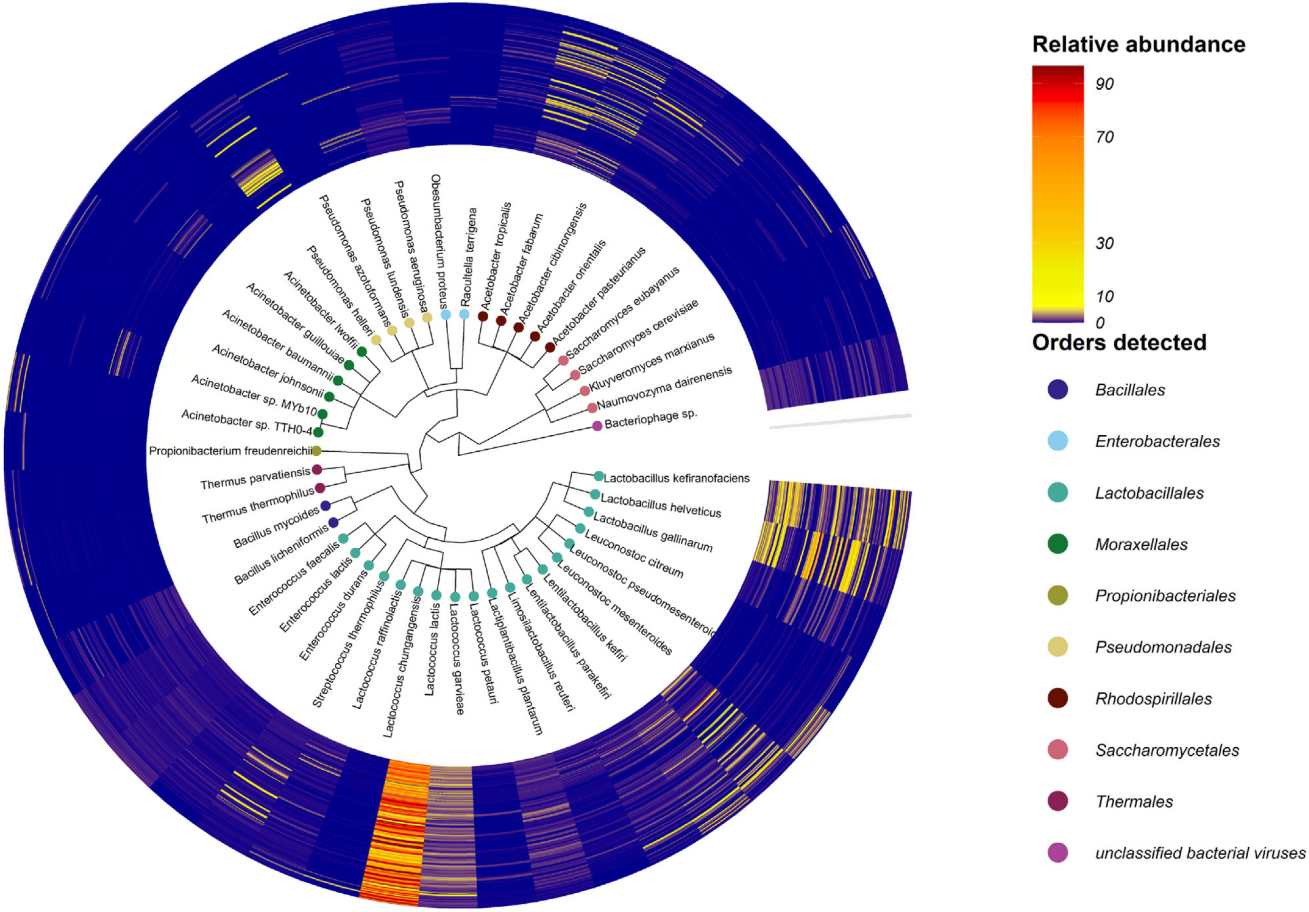
Detected Species and their relative abundance across milk kefir metagenomes Cladogram presenting a hierarchical overview of species detected in the kefir microbiome using the custom kraken2 pipeline. Tips represent the species detected, e.g., *Lc. lactis*, and the associated color of the tips describes the order of the species *Bacillales*, *Enterobacterales*, *Lactobacillales*, *Propionibacteriales*, *Pseudomonadales*, *Rhodospirillales*, *Saccharomycetales,**Thermales* and  Unclassified bacteriophage. The outer rings represent a circular heatmap displaying the relative abundance of each species across 256 metagenomes.

64 individual milk kefir grain samples sourced from 25 different countries (Figure S1) were used to carry out fermentations, in duplicate, to produce milk kefir from full fat pasteurized cow’s milk, with samples being collected after 8 and 24 h of fermentation for shotgun metagenomic analysis (Figure S2); yielding 108.7 GB of data (Table S1; Figure S2). The short read taxonomic profiler, Kraken2,^25^ was employed to determine the microbiome composition using a custom built Kraken2 database (Table S2). This identified 46 species of viral, eukaryotic and bacterial origin at different levels of relative abundance above a 1% threshold in the kefir metagenomics dataset (Figure 1; Table S3).

Of the 46 species, *Thermus thermophilus* and *Pseudomonas lundensis* were consistently detected in the control, unfermented milk samples, and thus not regarded as kefir-associated (Table S3). The yeast species *Saccharomyces eubayanus, Kluyveromyces marxianus*, and *Saccharomyces cerevisiae*, and combined bacteriophage were detected at low relative abundance (<2%) (Figure 1; Table S3). *Acetobacter cibinongensis, Acetobacter fabarum, Lactiplantibacillus plantarum, Lactobacillus gallinarum, Lactobacillus helveticus, Lactobacillus kefiranofaciens, Lactococcus garvieae, Lactococcus lactis, Lentilactobacillus kefiri, Lentilactobacillus parakefiri, Leuconostoc mesenteroides*, and *Pseudomonas helleri* were detected at ≥1% relative abundance in ≥10% of samples (the defined threshold of prevalence for this study) (Figure 1; Table S3). *Lc. lactis* was detected in 253 of the 256 samples at varying levels of relative abundance (2–96%). The three samples in which *Lc. lactis* was not detected at ≥1% relative abundance had been collected after 8 h of fermentation. Even in these instances, *Lc. lactis* accounted for sizable proportions of the relative abundance of the corresponding 24 h samples (16–78%) (Figure 1; Table S3).

**Figure 2:**
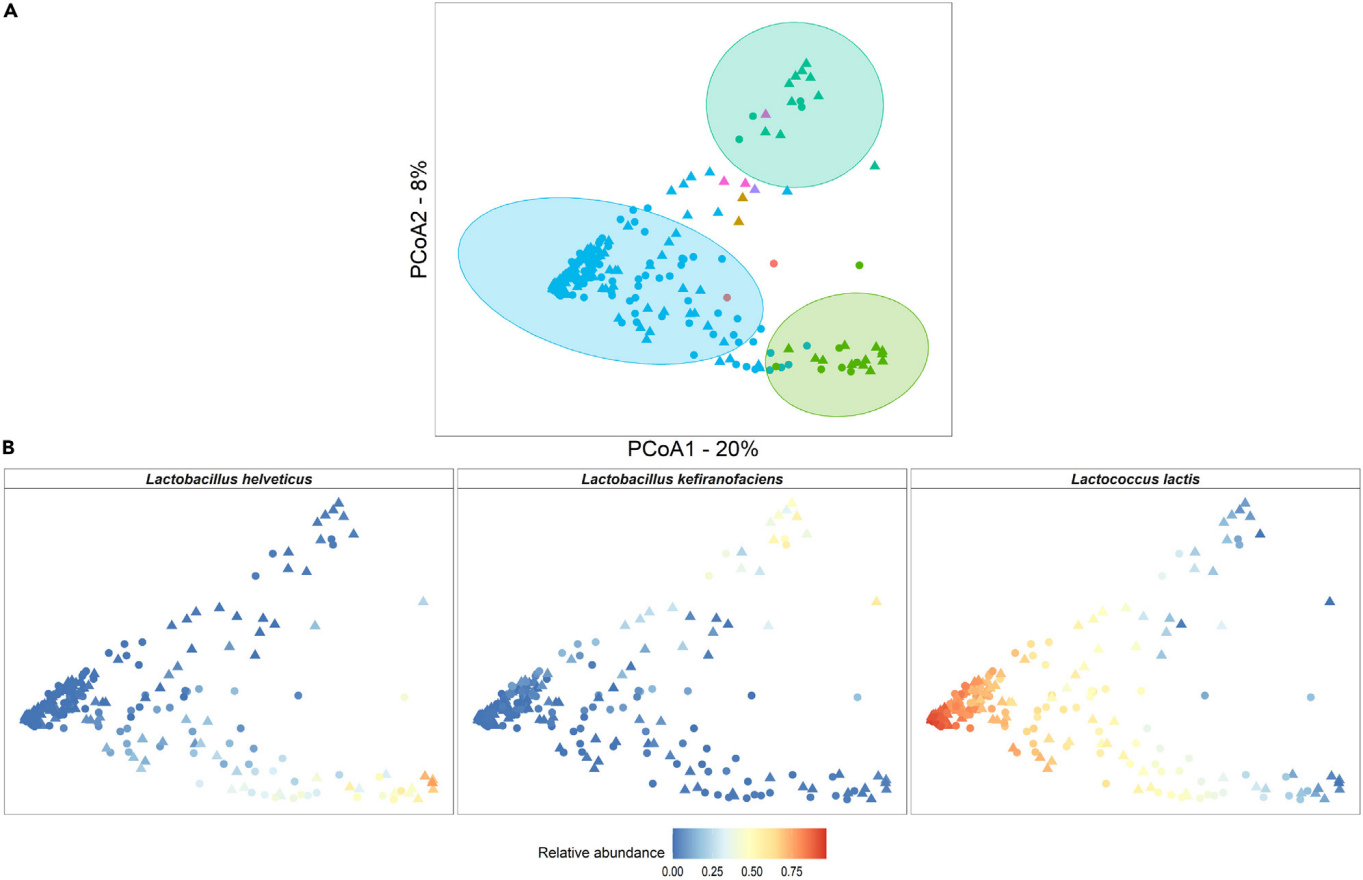
Variance in the compositional data among kefir metagenomes explained by the dominating microbial species (A) Principal coordinate analysis (PCoA) of beta diversity measured by Bray-Curtis dissimilarity of 256 kefir metagenomes, calculated for species-level composition. The PCoA plot displays the microbiome differences between kefir metagenomes dominated by different microbial species. Metagenomes are colored by the dominant species *A*. *orientalis,**Ac*. *sp.* TTHO-4, *L*. *helveticus*, *L*. *kefiranofaciens,**Lc. lactis,**Leuc*. *mesenteroides* and *P*. *helleri*. Metagenomes are shaped according to time point (8 h or  24 h). The 95% confidence ellipse is shown for metagenomes dominated by *Lc. lactis,**L. helveticus* and *L*. *kefiranofaciens*. (B) PCoA of beta diversity separated by the dominant species *L. helveticus*, *L. kefiranofaciens* and *Lc. lactis.* Metagenomes are colored by the relative abundance of the dominant species.

Overall, the kefir metagenomes were consistently dominated by one bacterial species (Figure 1; Figure 2; Table S3). This species was *Lc*. *lactis* in 215 samples (ranging in abundance values from 24.8 to 96.5%), *L*. *helveticus* in 19 samples (30.9–77%), *L*. *kefiranofaciens* in 14 samples (28–57.6%)*, Acetobacter orientalis* in 3 samples (41.5–65.6%), *Acinetobacter* sp. TTH0-4 in 2 samples (39.3–58.9%) and *Leuconostoc mesenteroides* in one sample (84.6%) (Figure 2A). There were no major changes in patterns of dominance between the 8 and 24 h time points for specific kefir fermentations in the majority of cases (i.e., n = 104 fermentations corresponding to 52 different starter grains), but notable changes were observed in 24 metagenomes corresponding to 12 grains. Specifically, metagenomes dominated by *P. helleri, Acinetobacter* sp. TTHO-4 and *Leuc*. *mesenteroides* did not persist as the fermentation process progressed to 24 h. LEfSe was used to identify differentially abundant taxa between the timepoints. *Pseudomonas* species and *T. thermophilus* tended to more abundant at 0 h (i.e., representing the microbiome of the milk substrate), *L. kefiranofaciens, Lentilactobacillus kefiri and L. helveticus*, tended to more abundant at 8 h whereas *Lc. lactis* tended to be more abundant at 24 h (Figure S3).

The permutational analysis of variance (PERMANOVA) statistical test revealed significant dissimilarity between kefir metagenomes, with the dominant microbial species accounting for the highest fraction of total variance (PERMANOVA: R2 = 0.36, p= <0.001). Pairwise PERMANOVA further confirmed significant differences between kefir metagenomes and revealed specific groupings that were significantly dissimilar, which included metagenomes dominated by *Lc. lactis*, *L. kefiranofaciens* and *L. helveticus* (Figure 2A). There were not enough metagenomes dominated by other prevalent microorganisms to be statistically regarded as additional subgroups. No dissimilarities were apparent when the influence of fermentation time point (Figure 2) or country of origin were considered.

### Phylogenetic analysis of assembled metagenomes identifies three putatively novel species in the kefir microbiome

**Figure 3:**
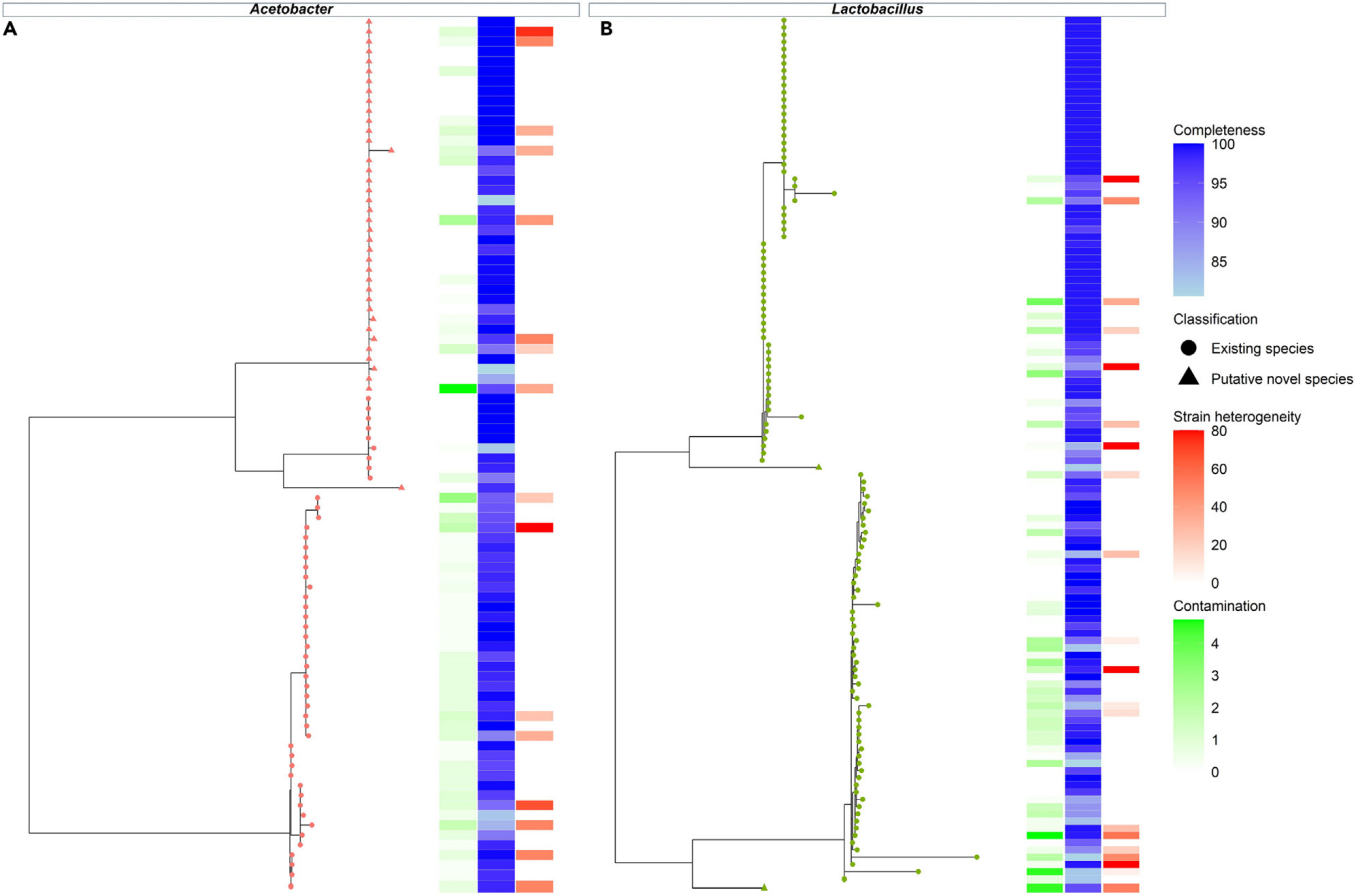
Putative new species of the genus *Acetobacter* and *Lactobacillus* within the kefir microbiome (A and B) Phylogenetic tree of high-quality MAGs of the genus A. *Acetobacter* and B. *Lactobacillus*, including putative new species. Outer band colored green, blue and red represent % contamination, completion and strain heterogeneity, respectively, as assessed using CheckM. The colored tip of the phylogram corresponds to the genus classification of the MAG. Genus classifications include *Acetobacter* and *Lactobacillus*. The tip shape of the phylogram indicates which MAGs are known species () or potentially novel species ().

In total, 718 MAGs with an estimated completion of ≥50% and ≤10% contamination were generated, and assigned to 18 different species (Figure S4; Table S4). Of these, 613 were high quality MAGs (over 80% complete, with less than 5% contamination) and corresponded to the species *A. fabarum*, *A*. *orientalis*, *Acinetobacter guillouiae*, *Acinetobacter albensis, L. helveticus*, *L*. *kefiranofaciens*, *Len*. *kefiri*, *Lc. lactis*, *Lc*. *raffinolactis*, *Leuc. mesenteroides*, *Leuc. pseudomesenteroides*, *P*. *helleri*, *Pseudomonas azotoformans*, and *S. thermophilus*. 41 MAGs had less than 95% average nucleotide identity (ANI) to the closest known NCBI prokaryote reference genome and thus may represent novel species. 39 of these MAGs are predicted to be representatives of the genus *Acetobacter*, with two belonging to the genus *Lactobacillus* (Figure 3). De-replication of the putatively novel species revealed that the MAGs assigned to the genus *Acetobacter* grouped together into the same species level bin, while the MAGs assigned to the genus *Lactobacillus* exhibited greater differences at the nucleotide level and grouped into separate clusters (Figure 3).

### Strain level phylogenetic analysis of the kefir microbiome uncovers strain subclusters and long-term strain-type retention

**Figure 4:**
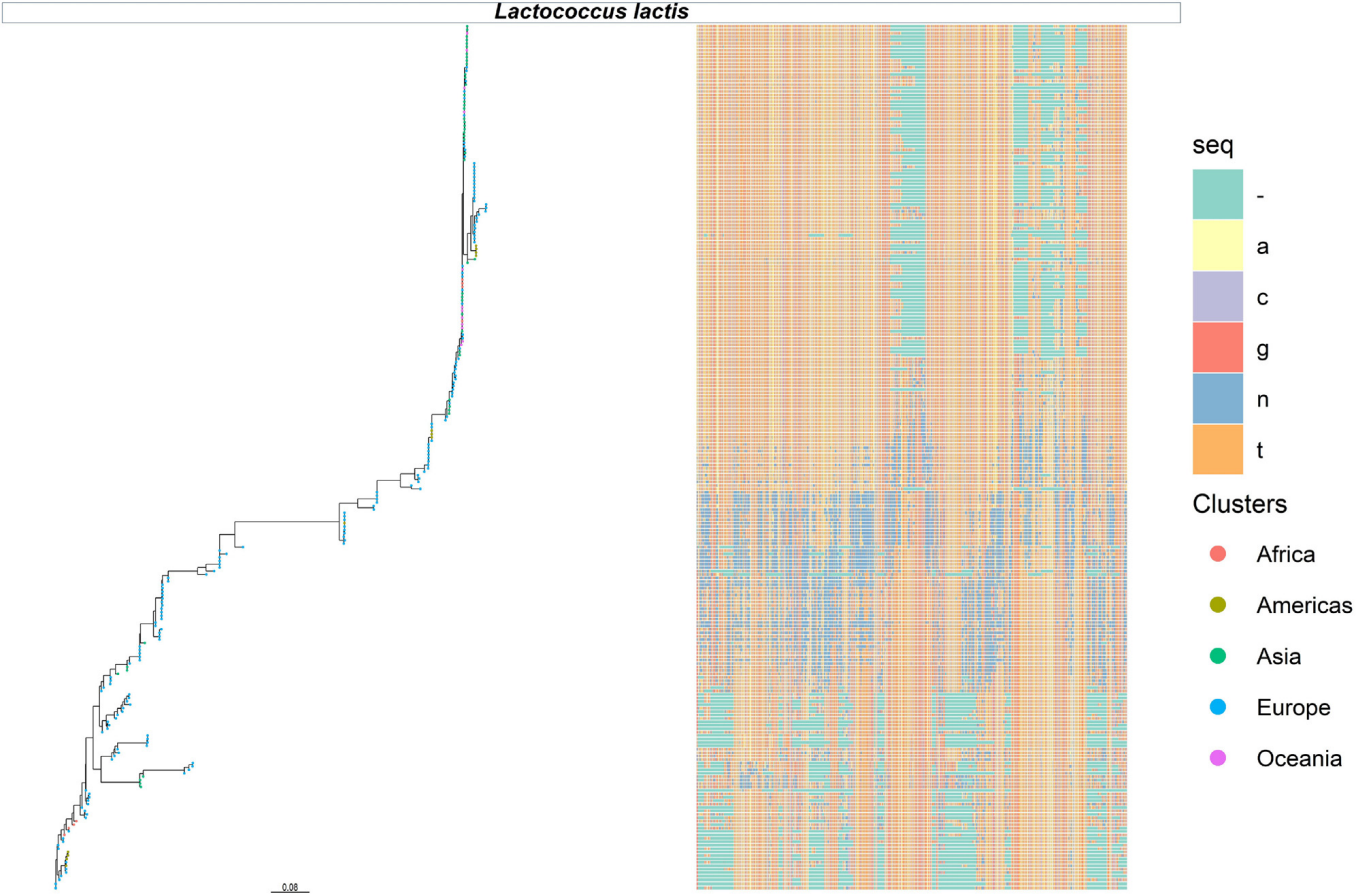
Strains of *Lc. lactis* strains detected across kefir metagenomes Strain level phylograms of *Lc. lactis* strains and corresponding multiple sequence alignment (MSA) files generated by StrainPhlAn 3. Colored tips of the phylogram corresponds to the time point of the kefir metagenome from which the strain was recovered. The MSAs contains completely specified nucleotides (A, C, G, T), ambiguous characters (N) and alignment gaps (–) inserted between the nucleotides of some of the sequences Other representative species phylograms are available in Figures S5‒S20.

StrainPhlAn 3 was chosen as the primary strain profiling tool in this study due to its sensitivity. StrainPhlAn 3 evaluates the alignment of marker genes to reference genomes to capture a sufficient portion of the reference genomes for variant calling.^26^^,^^27^ StrainPhlAn additionally reports the number of polymorphic sites across alignments. Sites are deemed polymorphic if they display a certain degree of variability in the base call that the dominant allele accounts for less than 80% of reads.^26^ StrainPhlAn 3 profiling of the 256-kefir metagenomes detected a total of 861 strains that represented 15 different species of bacterial origin and 3 different species of lactococcal phage (Figure 4; Figures S5‒S20; Table S5).

**Figure 5:**
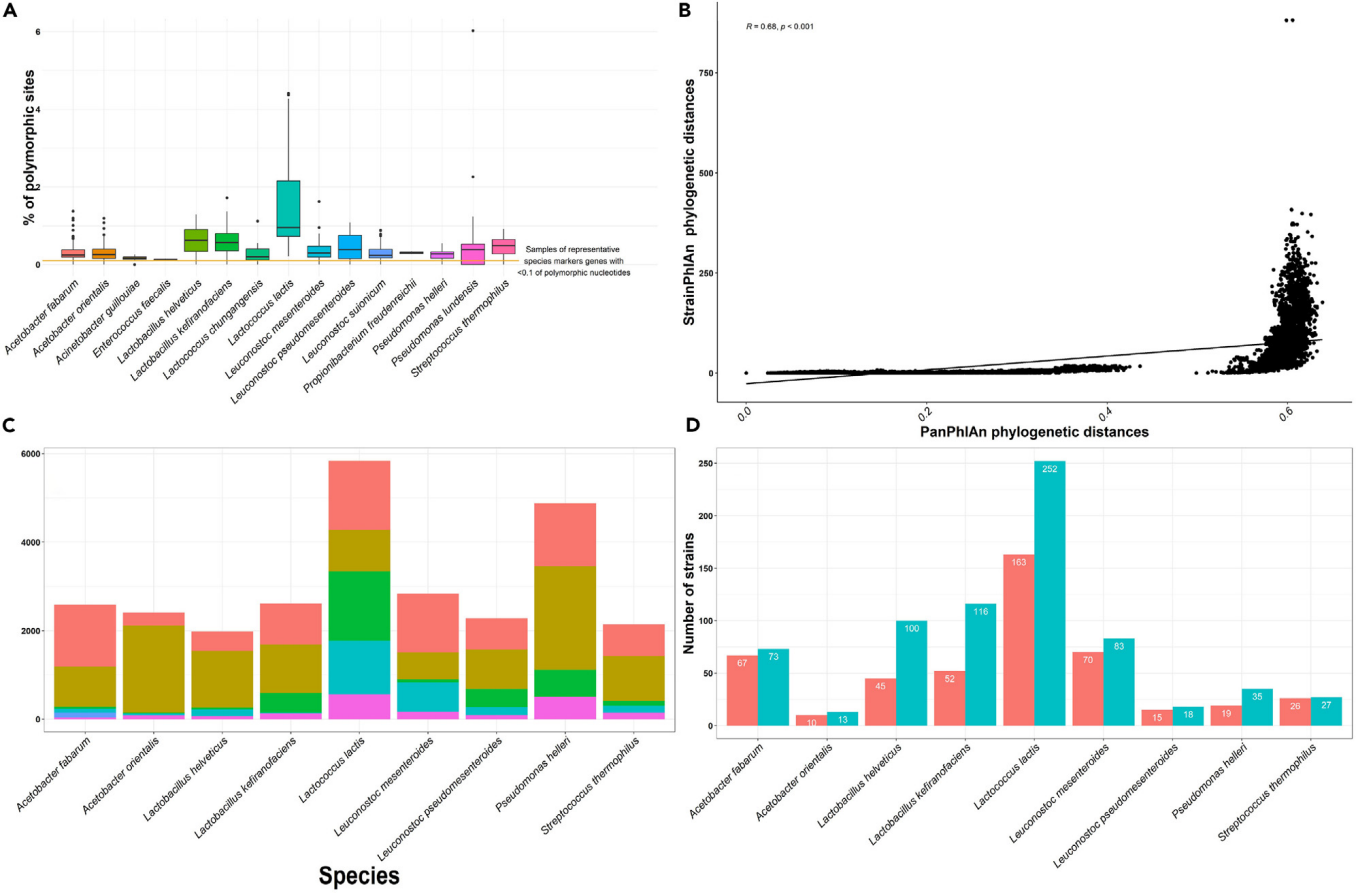
Analysis of representative strains, with joint identification by StrainPhlAn 3 and PanPhlAn 3 (A) Collective intra-sample strain diversity across metagenomes for each of the outlined 15 bacterial species (X axis). Each colored boxplot displays the range of polymorphic sites expressed as percentages (Y axis); along the MLSA of strains. (B) Graphical presentation of PanPhlAn 3 predicted gene families (Y axis) that compose the pangenome of the representative species (X axis). Details include number of gene families detected per pangenome and the number of gene families classified as  accessory,  core,  specific to cluster 1,  specific to cluster 2,  specific to cluster 3 and  strain specific. (C) Results of correlation analysis performed using the genetic distance between StrainPhlAn 3 (Y axis) and PanPhlAn 3 (X axis) predicted strains calculated using the Kendall correlation method. (D) Number of strains detected (Y axis) for each representative species (X axis) by the  StrainPhlAn 3 or  PanPhlAn 3 approach. Only representative species, with joint identification by StrainPhlAn 3 and PanPhlAn 3 were considered in this graph.

Intrasample strain diversity was evaluated across metagenomes, which established that >0.1 of nucleotides on detected marker genes for the examined species were polymorphic in 95% of metagenomes,^26^^,^^28^ consistent with the presence of multiple strains for the considered species across metagenomes. Examination of the strains of the species *A. fabarum*, *A. orientalis*, *Ac. guillouiae*, *E. faecalis*, *L. helveticus*, *L. kefiranofaciens*, *Lc. chungangensis*, *Leuc. mesenteroides*, *Leuc. pseudomesenteroides*, *Leuc. suionicum*, *Propionibacterium freudenreichii*, *P. helleri* and *S. thermophilus* revealed a percentage of polymorphic sites <2% suggesting the dominance of a single strain within each individual kefir metagenome. While strains of *Lc. lactis* (Figure 4) and *P*. *lundensis* (Figure S19) had a median percentage of polymorphic sites <2% in many cases, some specific metagenomes contained strains with a greater number of polymorphic rates, suggesting the possibility of multiple strains without single strain dominance in these metagenomes (Figure 5A; Table S5). Despite these exceptions, in general, the majority of the strain level diversity of a kefir metagenome is captured when one profiles the dominant strain present from each species.

As the metagenomics data collected was temporally ordered, an assessment of the succession patterns of strains was possible. Strains recovered from metagenomes produced from the same grain were compared using the p-distance evolutionary model to assess if the same strains were retained, or if lower abundance strains became dominant as the fermentation process progressed. Strains of *Pr*. *freudenreichii* derived from the same kefir grain varied substantially in nucleotide composition, differing at 40–60% of sites, indicating a strain succession event (Figure S17) may have occurred in the initial fermentations. No nucleotide differences were observed between strains from other species, including those derived from metagenomes with a higher rate of polymorphic sites, indicating that the strain of that species that dominated at 8 h continued to do so at 24 h.

In addition to comparing strains derived from the same kefir grain to uncover patterns of short term strain succession/retention, we also compared the phylogenetic distance between strains of the same species across all metagenomes using the Kimura 2-parameter distance evolutionary model. Firstly when considering variability, we observed large genetic distances between a number of strains of the species *Lc. lactis* (Figure 4), which were not observed for strains of the other, less abundant species examined. The large genetic distance between the *Lc. lactis* strains was further assessed using the p-distance evolutionary DNA model, which reported that a number of strains differed by ≥ 70% of sites, hindering downstream clustering analysis at the nucleotide level (Figure 4). For other species, PAM clustering analysis was used to identify the presence/absence of strain subclusters (Figures S5–S20). 2–4 distinct subclusters were predicted for the bacterial species *A. fabarum* (Figure S5), *A. orientalis* (Figure S6), *L. helveticus* (Figure S8), *L. kefiranofaciens* (Figure S9), *Leuc. mesenteroides* (Figure S14), *Leuc. pseudomesenteroides* (Figure S15), *Leuc. suionicum* (Figure S16), *P. helleri* (Figure S18) and *S. thermophilus* (Figure S20). Subclusters of these species contained strains with identical nucleotide composition along shared sites in the multiple sequence alignment and therefore were inferred to be of the same strain-type. Specifically, subcluster - 1 and - 2 of *A. fabarum* (Figure S5) and *Leuc. suionicum* (Figure S16), 1, 2 and 4 of *L. helveticus* (Figure S8), subcluster - 2 of *A. orientalis* (Figure S6) and *S. thermophilus* (Figure S20), subcluster - 1 of *L. kefiranofaciens* (Figure S9) and *Leuc. mesenteroides* (Figure S14) contained the same strain-types, despite the strains being derived from kefir grains sourced from different geographical locations.

**Figure 6:**
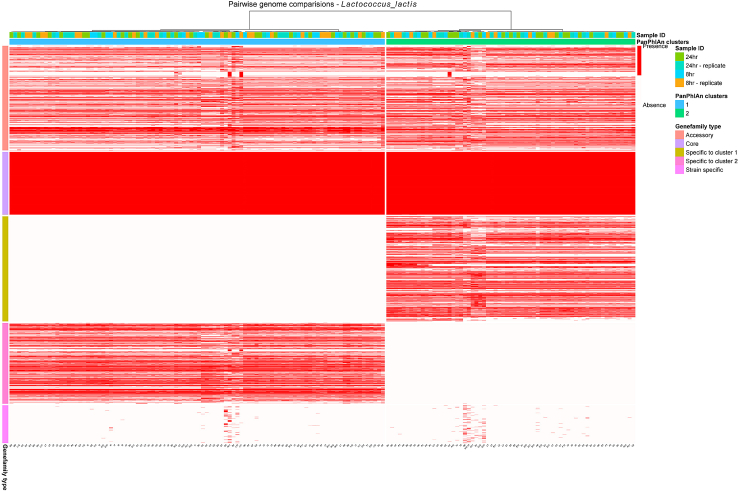
Pangenome of *Lc. lactis* strains detected within the milk kefir microbiome Representative heatmap representing the pangenome of *Lc. lactis* strains*;* rows represent PanPhlAn 3 detected strains and columns represent the UniRef.90 gene families identified per strain. Row side annotations display the grouping of each gene family, e.g., core gene or specific to cluster 1. Column side annotations display the assigned cluster of the detected strain according to the PanPhlAn 3 hierarchical clustering methodology (see STAR Methods) and the time point that the strain was recovered from.

To further investigate the variability between strains, we assessed the functional potential of strains through the application of a *meta*-pangenomic approach using PanPhlAn 3. PanPhlAn 3 evaluates the alignment of metagenomics reads to reference species pangenomes to capture genes and coverage information used to determine gene family presence/absence profiles per strain. PanPhlAn 3 was used to confirm and expand on information relating to subclusters predicted using the more sensitive StrainPhlAn 3 approach.^26^ This methodology facilitated the construction of pangenomes for the representative bacterial species *A. fabarum*, *A. orientalis*, *L. helveticus*, *L. kefiranofaciens*, *Lc. lactis*, *Leuc. mesenteroides*, *Leuc. pseudomesenteroides*, *P. helleri* and *S. thermophilus* (Figures 5B, 6, and S21‒S28).

Gene families within pangenomes could be grouped into three categories; ‘core’ if they were consistently recovered across strains, ‘accessory’ if they were identified in more than one strain and ‘strain specific’ if they were identified solely in one strain (Figures 5B, 6, and S21‒S28). Correlation analysis was performed using the genetic distance between StrainPhlAn 3 and PanPhlAn 3 strains. Positive linear correlations of medium to very high strength were consistently observed for strains for each of the nine representative species (Figure 5C) detected using both StrainPhlAn 3 and PanPhlAn 3. PanPhlAn 3 predicted strains were clustered using hierarchical clustering (see STAR Methods). Functional differences and hierarchical clustering between strains of *Lc. lactis* suggested the presence of two distinct subclusters. Hierarchical clustering typically agreed with the results of the PAM clustering methodology; however exact matching was not possible due to the detection of a different number of strains by both approaches (Figure 5D). As expected StrainPhlAn 3 consistently detected a higher number of strains (Figure 5D) than PanPhlAn 3, which in a small number of cases such as with respect to *L*. *helveticus* (45 vs. 100), prevented the confirmation and further functional analysis of subclusters 3 and 4. Similar results were observed for subclusters of *L. kefiranofaciens* and *A. orientalis* strains. As clustering results predicted using the PAM clustering methodology were mostly replicated using hierarchical clustering, we could further identify gene families unique to each subcluster and determine the proportions of the pan genome that these subclusters accounted for. Statistically significant functional differences between clusters was observed for the species *A. fabarum, L*. *helveticus, Lc. lactis, Leuc. mesenteroides, Leuc. pseudomesenteroides*, *P.* *helleri* and S*. thermophilus*, as determined using either the Mann-Whitney U test to compare two clusters, or the Kruskal-Wallis test to compare three clusters. Further assessment of the gene families unique to each subcluster revealed slight functional differences between subclusters of the species *A. fabarum* (Figure S21), *A. orientalis* (Figure S22)*, L*. *helveticus* (Figure S23) and *S. thermophilus* (Figure S28) with gene families unique to each cluster accounting for less than 10% of the species pangenome (Figure 5B). For all other species, sizable proportions of the pangenome were attributed to clusters (Figures 5B and S24‒S27). The *Lc. lactis* pangenome displayed the most variability, with 27% and 21% of the pangenome being assigned to cluster one and two, respectively (Figures 5B and 6), an observation that was consistent with the results of the StrainPhlAn 3 analysis. Gene family differences between clusters of *Leuc. mesenteroides* and *P. helleri* were consistent with the absence of gene families from one of the two clusters (Figures 5B, S25, and S27).

### The metabolic potential of kefir metagenomes separates by community type

As well as being used to investigate differences in the functional potential across strain-types, we further assessed functional potential across kefir metagenomes using HUMAnN 3.0, which led to the identification of 402 diverse metabolic pathways in the kefir microbiome. Pathways were grouped into 36 higher level classifications based MetaCyc (Metabolic Pathway Database)^29^ (Table S6). Many pathways were involved in biosynthesis, specifically for glycerol biosynthesis, glucose, ADP-L-*glycero*-beta-D-manno-heptose, chitin, starch, UDP-N-acetyl-D-galactosamine, CMP-3-deoxy-D-manno-octulosonate, colanic acid, mannose and UDP-N-acetyl-D-glucosamine. Fewer pathways were attributed to degradation. The carbohydrate degradation pathways detected suggest that the carbohydrates chitin, fructose, fucose, galactose, glucose, lactose, stachyose, starch, sucrose, trehalose, and xylose can be used by some kefir-associated microorganisms. Pathways involved in amino acid metabolism indicate that the amino acids L-glutamate, L-arginine, L-histidine, L-leucine, L-phenylalanine, L-tryptophan, L-valine, putrescine, 4-aminobutanoate and L-ornithine may be utilized during fermentation (Table S6). The number of metabolic pathways detected per sample ranged from 79 (M11) to 282 (F8) with a median value of 163 pathways. Separation was observed between the milk kefir samples at 8 h and 24 h and unfermented milk controls in terms of the prevalence of metabolic pathways. LEfSe detected 1, 59, and 89 differentially abundant pathways between 0, 8 and 24 h samples, respectively. Notably, we observed that pathways involved in carbohydrate metabolism and biosynthesis and unsaturated fatty acid biosynthesis were most abundant at 8 h, whereas those involved in amino acid biosynthesis cofactor, carrier, and vitamin biosynthesis and metabolism were most abundant at 24 h. Pathways were further grouped based on their presence across samples into the following 6 categories ‘core’ (n = 51) if they were present in 100% of metagenomes, ‘high’ (n = 105) if they were present in 70% but less than 100% of metagenomes, ‘moderate’ (n = 39) if they were present in 50% but less than 70%, of metagenomes, ‘low’ (n = 185) if they were present in multiple but less than 50% of metagenomes and ‘sample specific’ (n = 21). 51 pathways were deemed core pathways, as they were present in all kefir metagenomes. Notably, all core pathways detected could be attributed to the presence of *Lc. lactis*, as estimated using the per species metabolic pathway abundance output of HUMAnN 3.0.^26^ Additionally the relative abundance attributed to *Lc. lactis* typically accounted for the majority of the observed relative abundance per core pathway among metagenomes (Figure S29). Core pathways principally involved generic housekeeping functions such as carbohydrate metabolism and biosynthesis of amino acids (Table S6). Melonnpan^30^ was also used to predict the metabolites potentially produced by each kefir metagenome using the Uniref. 90 genefamiles identified by HUMAnN 3.0. The output was restricted to the top 10 most abundant metabolites in ≥10% of samples to ensure high accuracy. The top 10 metabolites predicted ordered according to their maximum relative abundance included the secondary bile acid, deoxycholic acid, glutamate, chenodeoxycholate and deoxycholate, cholate, xanthine, lithocholate, butyrate, and isobutyrate, nicotinic acid, cholestenone, and lithocholic acid.

**Figure 7:**
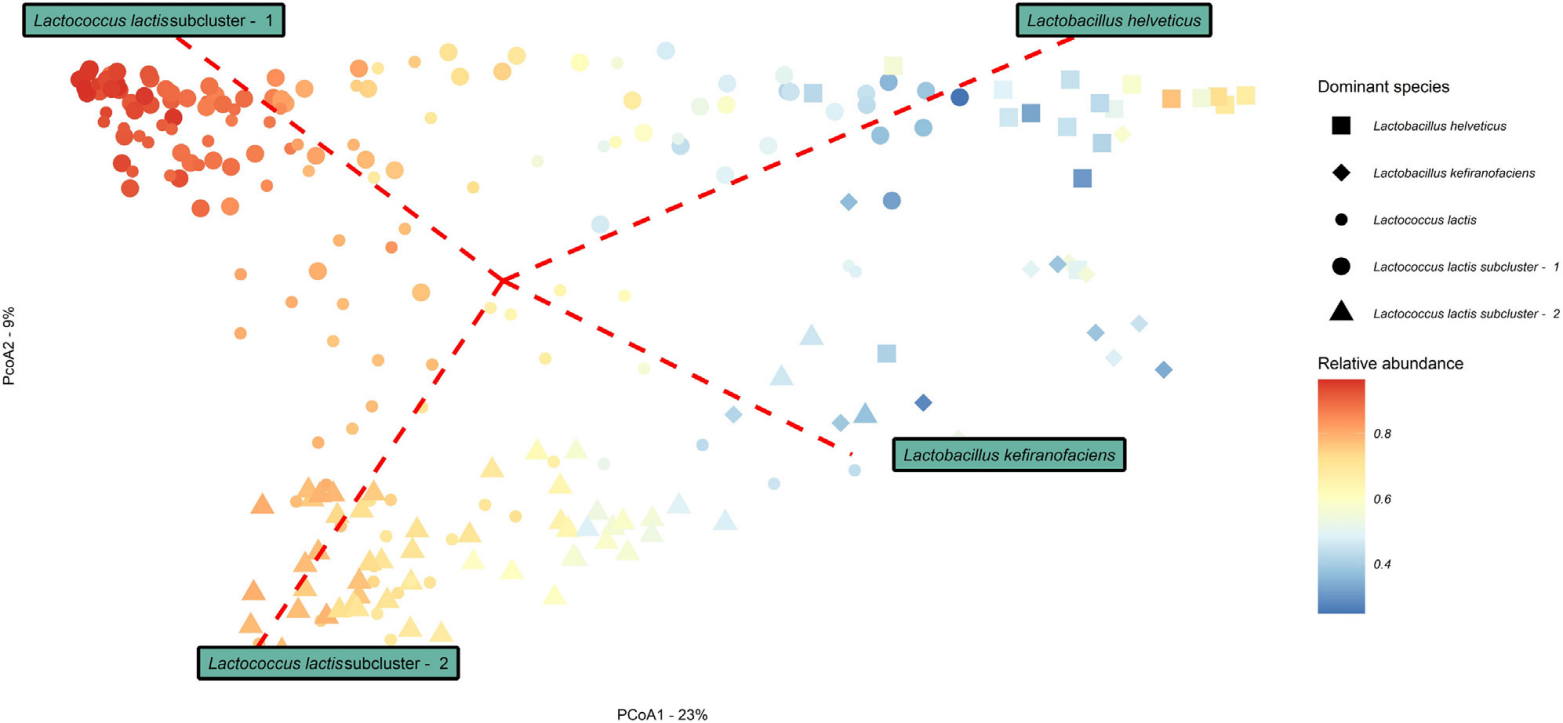
Variance in the metabolic potential among kefir metagenomes explained by the abundances of species/strains types Principal Coordinate analysis (PCoA) of beta diversity measure by Bray-Curtis dissimilarity for 256 kefir samples, calculated for metabolic composition as predicted using HUMAnN 3.0. Samples are shaped according the dominant species/strain subcluster (*Lc. lactis*, *Lc. lactis* subcluster - 1, *Lc. lactis* subcluster - 2, *L. helveticus* or *L*. *kefiranofaciens*). *Lc. lactis* represent kefir metagenomes in which complimentary strain profiling data was absent. Samples are colored by the relative abundance of the dominant species/strain subcluster.

Insights provided by strain subclustering together with species level taxonomic profiles were combined and utilized to provide insights into patterns of dissimilarity within the metabolic potential data among kefir metagenomes. Consistent with the dissimilar patterns identified in the composition data, we observed significant dissimilarity between the metabolic potential of kefir metagenomes, which can be explained when considering the dominant microbial species/strain type. Ordination clearly displayed the grouping of metagenomes by dominating microbial species/strain subclusters and relative abundance, in a pattern suggesting four distinct community types, extending the result obtained when considering the compositional data (see results section: Taxonomic profiling reveals distinct community types in the kefir microbiome). PERMANOVA (R2 = 0.5, p = 0.001) and Pairwise PERMANOVA further confirmed the significant differences between kefir metagenomes when considering the dominant microbial species/strain type (Figure 7).

### Functional comparisons of the representatives of each community-type reveals distinguishing metabolic and functional features

Further evidence of separation in metabolic potential between community types was provided by the identification of unique and differentially abundant metabolic pathways. LEfSe detected differentially abundant pathways between the community types, *Lc. lactis* subcluster - 1 (n = 24), *Lc. lactis* subcluster - 2 (n = 40), *L. helveticus* (n = 75) and *L. kefiranofaciens* (n = 80). When considering the present/absence of metabolic pathways across community types, all community types shared a collective of 222 metabolic pathways, while unique metabolic pathways were also observed in the community types *Lc*. *lactis* subcluster - 1 (n = 35), *Lc*. *lactis* subcluster - 2 (n = 16) and *L*. *helveticus* (n = 3). Differentially abundant pathways detected in the *Lc. lactis* subcluster - 1 community type included lactose and galactose degradation I, numerous vitamin biosynthesis pathways such as menaquinol (vitamin K-2), tetrahydrofolate (vitamin B9). Notably, of the 24 differentially abundant pathways, 5 of these produce tetrahydrofolate as the final product. Additionally, a number of amino acid biosynthesis pathways were enriched in this community type such as the superpathway of aromatic amino acid biosynthesis and other pathways involved in the production of the amino acids L-methionine, L-isoleucine and seleno amino acids. Similarly, the *Lc. lactis* subcluster - 2 community type included multiple differentially abundant biosynthesis pathways for the amino acids L-arginine, L-alanine, L-proline, L-isoleucine, L-valine, L-serine, L-threonine, L-homoserine, L-histidine and L-ornithine. Of note multiple pathways for glycolysis and L-arginine were detected for this community type. The *L. helveticus* community type contained differentially abundant pathways for inosine, sugar and galactose degradation, and pathways for L-rhamnose, inosine purines, L-lysine, L-glutamine, L-phenylalanine, L-tyrosine and L-tryptophan biosynthesis. Lastly the *L. kefiranofaciens* community type, contained differentially abundant biosynthesis pathways for ubiquinol, fatty acids, thiamine and the amino acids L-lysine, L-citrulline, L-arginine, L-threonine and L-methionine, L-ornithine.

**Figure 8:**
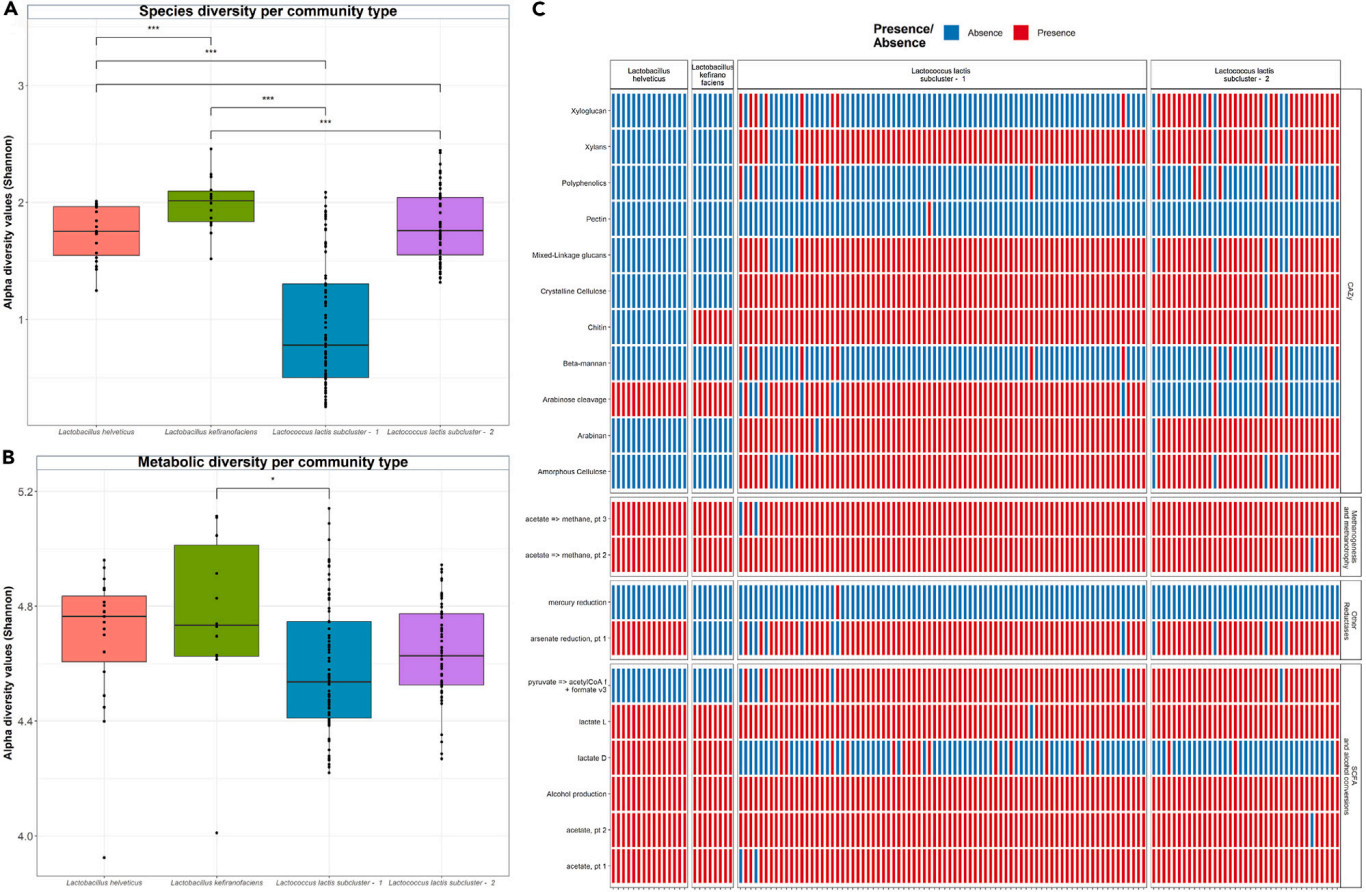
Diversity measures of community types within the kefir microbiome and characteristic metabolic function of *Lc. lactis* subcluster - 1, *Lc. lactis* subcluster - 2, *L. helveticus* and *L.* kefiranofaciens representative MAGs (A) Alpha diversity index observed for each community type when considering compositional data obtained using Kraken2. (B) Alpha diversity index observed for each community type when considering functional data obtained using HUMAnN 3.0. The coloring of the boxplots represents samples dominated by *L*. *helveticus*, *L. kefiranofaciens,**Lc. lactis* subcluster - 1 and *Lc. lactis* subcluster - 2. Pairwise tests were carried out between the dominant microbial species/strain using the Dunn’s test for nonparametric data. Corrected p values indicated as p < 0.05, ∗; p < 0.01, ∗∗; p < 0.001, ∗∗∗; p < 0.0001. ∗∗∗∗. (C) Presence/absence of annotations indicative of characteristic metabolic functions involved in catalyzing carbohydrate degradation (CAZymes), methanogenesis and methanotrophy, other reductases and short chain fatty acids and alcohol conversions (Y axis) across representative MAGs identified using dRep and derived from the community types *Lc. lactis* subcluster - 1, *Lc. lactis* subcluster - 2, *L*. *helveticus* and *L.* kefiranofaciens (X axis). The color of each cell of the heatmap represents the presence  or absence  of annotations.

Within-sample diversity at the species and metabolic level were compared across samples representing the four distinct community types using alpha diversity measures (Figures 8A and 8B). Samples corresponding to *Lc*. *lactis* subcluster - 1 exhibit significantly lower species alpha diversity compared to the other community types (p= <0.001) (Kruskal-Wallis multiple comparison). *L. helveticus* and *Lc. lactis* subcluster - 2 dominated samples exhibit significantly higher alpha diversity than the *L. kefiranofaciens*-dominated samples (p= <0.001) and *Lc*. *lactis* subcluster - 1 dominated samples (p= <0.001) (Kruskal-Wallis multiple comparison) (Figure 8A). When considering metabolic diversity, samples corresponding to *Lc*. *lactis* subcluster - 1 exhibit significantly lower alpha diversity compared to the *L. kefiranofaciens*-dominated samples (p= <0.001) (Kruskal-Wallis multiple comparison) (Figure 8B). As many of the differentially abundant pathways identified in each community type related to metabolisms, we selected high quality MAGs, representing the dominant microbial species in each community type, to compare functional annotations encoding metabolic traits. Selection was based on superior quality scores and included >80% completion, <5% contamination and 0% strain heterogeneity as assessed using CheckM. This revealed the potential capacity of MAGs representing the dominant microbial species in each community types to produce alcohol and produce the short chain fatty acid (SCFA) acetate. L-lactate production was prevalent across MAGs with only one exception occurring in the *Lc. lactis* subclusters - 1. *Lc. lactis* subclusters were further able to metabolize xylans, mixed-linkage glucans, cellulose, and arabinan (Figure 8C). *Lc. lactis* subclusters also contained annotations supporting the complete biosynthetic pathways of the amino acids cysteine, valine, isoleucine, leucine, arginine, proline, 2-oxoisocaproate and histidine. The *L.* *helveticus* and *L. kefiranofaciens* representatives, contained no biosynthetic pathways and few CAZY annotations, with annotations supporting arabinose cleavage in both sets of MAGs and chitin degradation in *L. kefiranofaciens* (Figure 8C). A number of genes were highly prevalent and solely identified in MAGs of *Lc. lactis* subclusters - 1, which encoded for components of the pathways Puu, reported for the production of the neurotransmitter γ-aminobutyric acid (GABA).^31^ Specifically, the identified genes included the *puuD* gene encoding for a γ-Glu-GABA hydrolase, which plays a role in the Puu pathway by converting γ-Glu-GABA into GABA^32^ and the genes *gadB* and *gadC* encoding for glutamate decarboxylases (GadB) and Glu/GABA antiporter, respectively (Figure 4). The glutamic acid decarboxylase (GAD) system provides a full mechanism by which *Lc. lactis* subclusters - 1 can produce GABA. Additional genes encoding for the arginine deiminase system were further detected. Genes supporting the exopolysaccharide production protein ExoZ and ExoY, were commonly detected in MAGs of *L. helveticus* and *L. kefiranofaciens*, and undetected across MAGs of *Lc. lactis* subclusters, except for the detection of genes supporting ExoY in one representative MAG of *Lc. lactis* subclusters - 2.

Finally, we have harnessed previous research by Walsh et al. 2016, whereby paired metagenomics and metabolomics data were used to reveal strong associations between the production of specific metabolites with particular kefir-associated species. More specifically, here we examined our data to identify metabolic features that may explain these associations. For example the metabolites phenylethyl alcohol (R = 0.6 and p = 0.04) and ethyl acetate (R = 0.52 and p = 0.05) associated with the presence of *L. kefiranofaciens*, with supportive annotations including the presence of genes encoding for the enzymes with aryl-alcohol dehydrogenase and acetyl transferases activity, respectively, across *L. kefiranofaciens* MAGs.^6^

### Phylogenetic placement of representatives of each community-type provides taxonomic classification and supports the elevation of representatives of *Lc. lactis* subcluster - 2 to a distinct species

**Figure 9:**
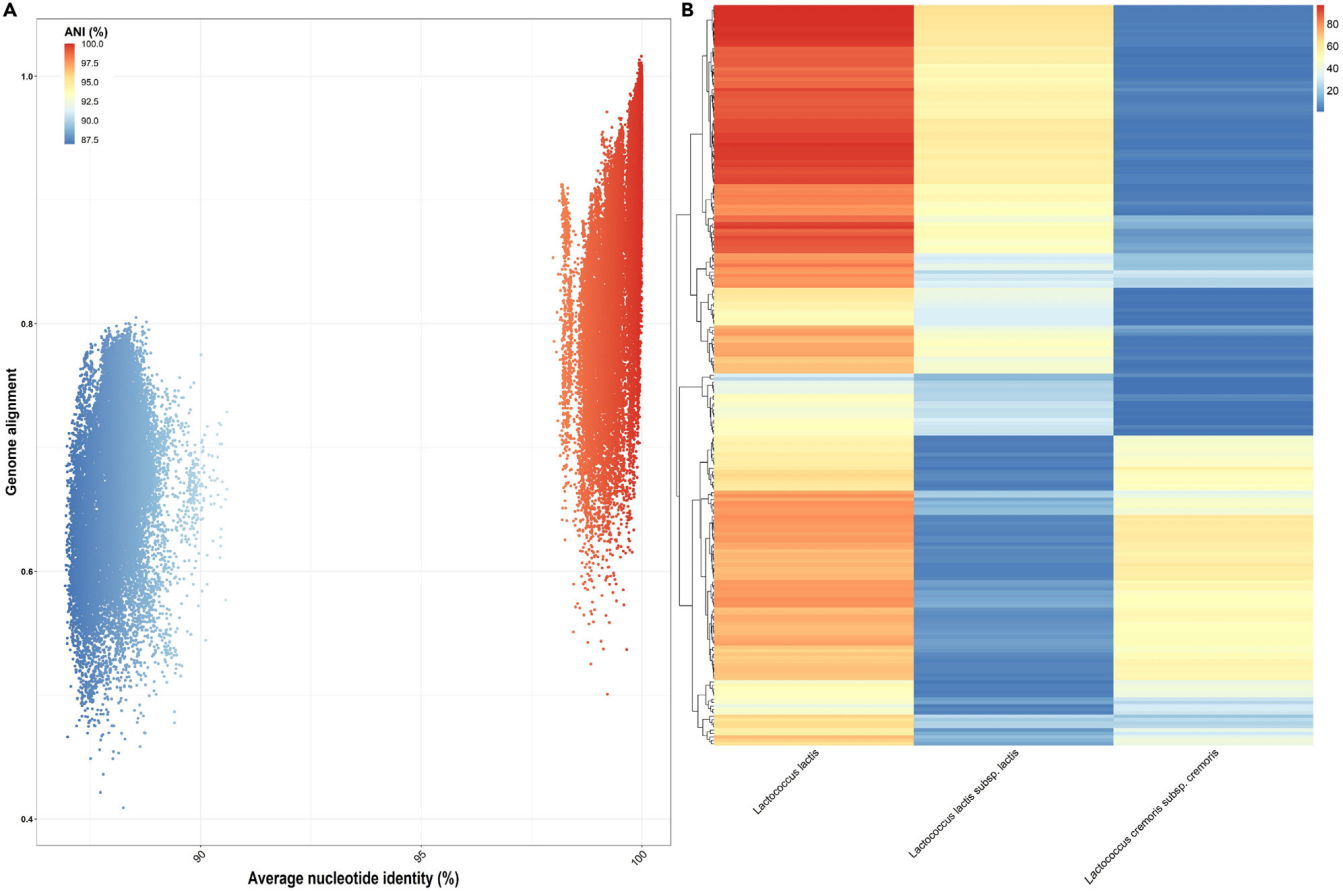
Separation of *Lc. lactis* subclusters based on Average nucleotide identity (ANI) and relative abundance across kefir metagenomes (A) ANI gaps exist near ∼91% ANI, between representatives of *Lc. lactis* subcluster - 1 and *Lc. lactis* subcluster - 2. Each dot represents the ANI (X axis) and genome alignment percentage values (Y axis) resulting from pairwise comparison within and between subclusters. Colors represent the ANI values. (B) Heatmap displaying the relative abundance across 256 metagenomes of the species *Lc. lactis*, predicted using Kraken2, and *Lc. lactis* subsp. *lactis* and *Lc. cremoris* subsp. *cremoris* both predicted using MetaCache.

High quality MAGs, representing the dominant microbial species in each community type, were further analyzed to identify closely related reference genomes. The L. *helveticus* representatives were mostly related to the reference genome GCF_000160855.1 with ANI values within the 97% range. The L. *kefiranofaciens* representatives were most related to the reference genome GCF_900103655.1, which represents the sub-species *L. kefiranofaciens* subsp. *kefiranofaciens* with ANI values within the 99% range and as such were deemed closely related genomes. The *Lc. lactis* subcluster - 1 MAGs were mostly related to the reference genome GCF_900099625.1, which is a representative of the sub-species *Lc. lactis* subsp*. lactis*, with ANI values distributed within the 98% range. The *Lc. lactis* subcluster - 2 MAGs were mostly related to the reference genome GCF_002078765.2, which is a representative of the sub-species *Lc. lactis* subsp*. cremoris* with ANI values distributed within the 97–99% range. As a recent study has proposed the elevation of *Lc*. *lactis* subsp*. cremoris* to the species level as *Lc. cremoris*,^33^ we performed pairwise genome comparisons using dRep between MAGs of the *Lc. lactis* subclusters. ANI values, between MAGs of *Lc. lactis* subcluster - 1 and *Lc. lactis* subcluster - 2 were lower than 95% ANI (Figure 9A), which generally represents a threshold for species delimitation.^34^ As *Lc. cremoris* has yet to be validated under the rules of the International Code of Nomenclature of Prokaryotes^35^ many bioinformatic tools, including those employed in this study, will not have adjusted their databases accordingly. Therefore, we re-evaluated the prevalence and relative abundance of *Lc. lactis* predicted using Kraken 2, which may have provided overestimations solely for the identification of the species *Lc. lactis*. Initially, when using Kraken 2, *Lc*. *lactis* dominated in 215 samples (ranging in abundance values from 24.8 to 96.5%), now through the application of MetaCache, we determined *Lc*. *lactis*, specifically *Lc. lactis* subsp*. lactis*, dominated in 125 samples (ranging in abundance values from 16 to 63%) and *Lc. cremoris*, specifically *Lc. cremoris* subsp*. cremoris*, dominated in 90 samples (ranging in abundance values from 23 to 60%). Furthermore, *Lc. lactis* subsp*. lactis* and *Lc. cremoris* subsp*. cremoris* were detected across all kefir metagenomes. We further confirmed the dominance of sub-species *L*. *kefiranofaciens* subsp*. kefiranofaciens* across metagenomes compared to the *L. kefiranofaciens* subsp*. kefirgranum* sub-species, the latter accounting for just 0–0.001% relative abundance across metagenomes.

### Virus identification highlights the prevalence of phages in kefir fermentations

Short read taxonomic profiling consistently detected the presence of bacteriophage at low relative abundance across samples, but was unable to assign the reads to specific species (Figure 1). Subsequent strain level profiling detected *Lc.* phage bIL285 (Figure S11), *Lc*. phage bIL309 (Figure S12) and *Lc.* phage bIL310 (Figure S13), but was unable to detect additional bacteriophage or provide associated functional annotations for the detected strains*.* Putative lysogenic viral contigs were consistently recovered in all high-quality MAGs constructed in this study. The level of phage detected was consistent with phage infection being common across all predominant species detected in the kefir metagenomes considered in this study, where one to a maximum of two prophage were identified per MAG. Additional viral sequences were also obtained by inspection of assemblies generated for viral features using the empirical screening criteria. Viral contigs may be representatives of prophage and/or free phage. Viral contigs were assessed for the presence of virally encoded auxiliary metabolic genes (AMGs) using DRAM-v.^36^ AMGs represent a unique category of viral genes that share homology with host metabolic genes but are adapted in viral genomes and function to reconfigure the metabolism of the host often to benefit viral synthesis.^37^ The DRAM-v analysis detected 65 potential AMGs such as *pstS* and *phoU* across the kefir metagenomics dataset, 26 and 39 of which were deemed high (auxiliary scores 1–2) and medium-ranked (auxiliary scores 3) confidence AMGs, respectively. Notably, no AMGs were recovered from milk samples. Annotations associated with these genes suggests that kefir-associated bacteriophage encode AMGs that could play roles in host energy generation, carbohydrate utilization and organic nitrogen transformation, transport and other miscellaneous functions. Among the genes detected was *pstS*, which encodes a phosphate transport system substrate-binding protein which may function to redirect phosphate acquisition for phage genome replication. Furthermore, the presence of *phoU* may enable rapid uptake of free phosphate for use in DNA synthesis^38^ to increase viral production.

## Discussion

In this study we applied metagenomics-based identification and characterization of the microorganisms present in milk kefir produced by milk kefir grains from different sources to investigate the pan-metagenome in milk kefir fermentations. A detailed knowledge of the pan-metagenome within milk kefir, provides considerable insights into the heterogeneity of these populations, while also uncovering highly prevalent and key core or conserved features, which is of commercial and scientific value. In line with Ibacache-Quiroga et al., 2022, this study found that, for the fermentation substrate and conditions employed, Eukarya represented less than 2% of the kefir microbiome, with the majority of the microbiota attributed to bacterial taxa. We further build on previous publications, which determined the continued occurrence and dominance and of *L. kefiranofaciens* and *Len. kefiri* within the less diverse milk kefir grain microbiome.^5^ Specifically species, coupled with strain, level analysis revealed that one microbial strain, most frequently strains of the sub-species *Lc. lactis* subsp*. lactis* or *Lc. lactis* subsp*. cremoris* (named under the current International Code of Nomenclature of Prokaryotes), tended to dominate in the individual kefir (Figure 9). Other strains that dominated were representative of the sub-species *L*. *kefiranofaciens* subsp*. kefiranofaciens* and the species *L*. *helveticus* (Figures 1, 2, and 7). Both short read taxonomic profiling and phylogenetic placement of MAGs could not assign strains of *L.* *helveticus* to the sub-species level. We note that in the majority of the fermentations analyzed (n = 104 of the 128) the pattern of dominance remained stable between the microbiome of 8 and 24 h fermentations, suggesting stability in the kefir ecosystem as the fermentation process progresses (Figure S3).

The observed pattern of dominance was the principle factor driving the separation of kefir metagenomes into four distinct community types, labeled as *Lc. lactis -* subcluster - 1 (*Lc. lactis* subsp*. lactis*)*, Lc. lactis -* subcluster - 2 (*Lc. lactis subsp. cremoris*), *L*. *helveticus* and *L. kefiranofaciens* (*L*. *kefiranofaciens* subsp*. kefiranofaciens*) (Figure 7). We provide further evidence to support the reclassification of sub-species *Lc. lactis* subsp*. cremoris* to the distinct species, *Lc. cremoris*^33^ through the pairwise genome comparisons of high-quality representative MAGs. It has been shown that a number of phenotypic features differentiate between *Lc. lactis* and *Lc. cremoris*, such as arginine utilization, maltose utilization, growth temperature, salt tolerance, production of ammonia from arginine via the arginine deiminase system and GABA production.^39^ Here we further confirm some of the aforementioned features at the genotypic level, including the presence of the entire complement of gene annotations supporting GABA production and the production of ammonia, uniquely detected in the *Lc. lactis -* subcluster - 1 representatives. Even though not always dominant, *Lc. lactis* and *Lc. cremoris* were detected in all samples at varying levels of relative abundance (Figure 9B), providing evidence that they constitute part of the core microbiome in kefir fermentations. The identification of a core microbiome, community patterns and highly prevalent microbial species in kefir fermentations helps to define the core pan-metagenome and determine the minimal compositional framework for a fermented milk product to be considered as a kefir.

The genetic relationship between representatives of *Lc. lactis* subsp*. lactis* and *Lc. lactis* subsp*. cremoris* (∼91% ANI) (Figure 9A) is in line with Li et al.,^33^ and further supports the elevation of *Lc. lactis subsp. cremoris* to the species level as *Lc. cremoris*.^33^ Such insights are particularly noteworthy as most bioinformatic tools available, including those implemented here, failed to delineate between *L*c*. lactis* and the proposed *Lc. cremoris* at the species and strain level. As a result of both species being consistently detected in the kefir metagenome and often accounting for a high relative abundance, previous studies concerning kefir may have overrepresented the abundance of *Lc. lactis*.

Species sub-structures were observed for 11 microbial species (Figures 6, S5‒S9, S12–S16, S18, and S20). Short term strain retention was apparent in repeat fermentations with only one apparent instance of a different strain from the same species becoming dominant as the fermentation progressed. We also provide evidence of long-term strain-type retention through evolutionary comparisons between strains of the same secondary subcluster using the Kimura 2-parameter model. Such evolutionary comparisons identified the presence of the same strain-type, defined using the stringent threshold of identical nucleotide compositions in reconstructed marker genes, in kefirs derived from grains sourced from different geographical locations. Strain level identification was complemented by an in-depth assessment of functional differences between strains derived from the kefir ecosystem (Figures 5B, 6, and S21‒S28). Assessment at the functional level further confirmed a number of species sub-structures (Figures 5 and 7) and revealed few functional intra-species differences between strains of *A. fabarum* (Figure S21), *A. orientalis* (Figure S22), *L*. *helveticus* (Figure S23) and *S. thermophilu*s (Figure S28). The detection of few functional differences suggests the presence of strains derived from the same sub-species. However, for other representative species such as *Leuc. mesenteroides* (Figure S25), we identified notable differences across the recovered strains that could be attributed to the presence/absence of cluster specific gene families, and identified shared and unique gene families between strains of *Lc. lactis* and *Lc. cremoris*. The identification of a species pangenome and the grouping of gene families according to their presence/absence in strains provides a novel insight into the gene catalog of kefir-associated strains and can inform future efforts to assess sub-species diversity.

The 222 metabolic pathways shared by the different community types may help to explain why some kefir fermentations that are dominated by different species/strains can have similar characteristics such as flavor and smell.^40^ Furthermore the detection of 51 metabolic pathways across all kefir metagenomes considered in this study and 105 highly prevalent pathways provides a means to go beyond taxonomy to define the minimal functional framework for a fermented milk product to be considered as a kefir MelonnPan predicted a number of metabolites to be abundant and prevalent across kefir samples, including the metabolites deoxycholic acid and GABA, of interest as an neurotransmitter,^41^ respectively. We further identify potentially supportive genome annotations for a number of kefir derived species and associated metabolic compounds made in previous publications. Annotations consistent with the ability to produce of ethyl acetate in *L. kefiranofaciens* are particularly noteworthy as GC-MS analysis revealed a 59.15% increase in this metabolite in a kefir milk, as a result of a spike in with the strain *L. kefiranofaciens* NCFB 2797.^6^ For further details concerning the volatile compounds produced by milk kefir see.^5^^,^^6^^,^^42^ Our analysis also provided insights into the identification and functional annotation of viral sequences, resulting in the metabolic categorization of putative AMGs, such as *pstS* and *phoU*, found across kefir metagenomes as thus representative of the accessory pan-metagenome of milk kefir. The presence of these phage-associated genes adds to the paradigm that phosphate scavenging is critical to phosphate-intensive viral reproduction.^43^^,^^44^ Such analysis highlights the presence of certain viral species and strains and provides insights into the functional potential of the viral component of the kefir microbiome.

Ultimately, this study provides the most comprehensive insight into the microbiomes of kefir to date through the establishment of a pan-metagenome, yielding novel information into the species, including putative new species and those residual of the unfermented milk, strain variability and potential functionality that can occur in kefir microbiomes derived from kefir grains sourced from across the globe. Such insights extend public knowledge of the microbial species present in the artisanal beverage, which can inform further guidelines on kefir production and help to raise awareness of the microbial species and metabolic pathways commonly found in the milk kefir microbiome. The insights and workflows outlined in this manuscript could also be applied to other fermented foods to help shape future regulatory guidelines relating to the microbial content of specific foods.

### Limitations of the study

As outlined in Walsh et al., at present, there is a need for additional eukaryotic reference genomes to reflect the eukaryotic species detected using culture based approaches.^45^ This coupled with the low relative abundance of eukaryotic species within the milk kefir microbiome, influences the reporting capacity of shotgun metagenomics analysis and, as such, this study may have failed to detect eukaryotic species present in the milk kefir microbiome.

## STAR★Methods

### Key resources table

**Table:** 

REAGENT or RESOURCE	SOURCE	IDENTIFIER
**Biological samples**		

Milk kefir liquid samples	This study	N/A

**Critical commercial assays**		

DNeasy PowerSoil Pro Kits	Qiagen	Cat# 47014
Nextera XT DNA Library Preparation Kit	Illumina	FC-131-1096
Nextera XT Index Kit v2, Set A/D	Illumina	FC-131-2001/4
Agilent High Sensitivity DNA Kit	Agilent Technologies, Inc.	DNF- 5067-4626

**Deposited data**		

Sequencing data	This study	ENA Project: PRJEB65292

**Software and algorithms**		

Bowtie2	Bowtie2	https://github.com/BenLangmead/bowtie2
Trimgalore	https://github.com/FelixKrueger/TrimGalore	https://github.com/FelixKrueger/TrimGalore
Kraken 2	(Wood et al.^25^)	https://github.com/DerrickWood/kraken2
Bracken	(Lu et al.^46^)	https://github.com/jenniferlu717/Bracken
HUMAnN 3	(Beghini et al.^47^)	https://huttenhower.sph.harvard.edu/humann/
Melonnpan	(Mallick et al. 33^30^)	https://huttenhower.sph.harvard.edu/melonnpan/
MEGAHIT	(Li et al.^48^)	https://github.com/voutcn/megahit
MetaWRAP	(Uritskiy et al.^55^	https://github.com/bxlab/metaWRAP
CheckM	(Parks et al.^49^)	https://github.com/Ecogenomics/CheckM
GTDB-Tk	(Chaumeil et al.^50^)	https://github.com/Ecogenomics/GTDBTk
dRep	(Olm et al.^51^)	https://github.com/MrOlm/drep
DRAM	(Shaffer et al.^36^)	https://github.com/WrightonLabCSU/DRAM
PanPhlAn 3	(Asnicar et al.^52^)	https://github.com/SegataLab/panphlan
StrainPhlAn 3	(Truong et al.^28^).	http://segatalab.cibio.unitn.it/tools/strainphlan/
Metacache	(Müller et al.^53^)	https://github.com/muellan/metacache
VirSorter2	(Guo et al.^54^)	https://github.com/jiarong/VirSorter2
R version 4.1.2	R Core Team	https://www.r-project.org

### Resource availability

#### Lead contact

Further information and requests for resources and reagents should be directed to and will be fulfilled by the lead contact, Paul Cotter (paul.cotter@teagasc.ie).

#### Materials availability

This study did not generate new reagents.

### Method details

#### Sample collection, preparation, pre-processing and DNA extraction

64 milk kefir grains and two milk kefir samples were obtained from 25 different countries (Figure S1). Samples were donated or purchased from private households that consented to their use in the study. Upon arrival, the grains were weighed and divided in two. Half of the divided grain was frozen immediately at -20°C and the other half was cultured continuously for one to two weeks until the grains reached 4 grams in weight. After this growth period, each kefir grain was cultured twice by inoculating the grains into 200 ml of full fat cow’s milk (Avonmore, Ireland; 2 % wt/vol) in a laminar flow hood and placing in an orbital shaker incubator at 24°C. 20 ml fermented milk was collected after 8 hours (hr) and 24 hr of the fermentation process for each of the successive fermentations. In total, four samples were collected for each fermentation initiated by each of the 64 kefir grains sourced in this study, resulting in a total of 256 kefir samples. Following collection, the kefir milk samples were stored at −20°C prior to DNA extraction, where 15 ml milk was used. Non-fermented full fat cow’s milk samples were used as controls (Figure S2).

Prior to DNA extraction, 15 ml of kefir milk was centrifuged at 5,444 × g for 30 minutes (min) at 4°C. Samples were washed with 10 ml of phosphate buffered saline (PBS) and centrifuged again at 5,444 × g for 15 min, to remove protein and fat residues. Two additional rounds of washing and centrifugation were performed before pelleting the microbial cells. The cell pellet was resuspended in 800 μl of Solution CD1 from the DNeasy PowerSoil Pro Kits. Total DNA from the resuspended pellets was extracted and purified according to the standard DNeasy PowerSoil Pro Kits kit protocol.

#### Preparation of libraries, shotgun metagenomic sequencing and data pre-processing

Total DNA was initially quantified and qualified by gel electrophoresis and using the NanoDrop 1000 (BioSciences, Dublin, Ireland) prior to more accurate quantification with the Qubit High Sensitivity DNA kit (BioSciences, Dublin, Ireland). Whole-metagenome shotgun libraries were prepared in accordance with the Nextera XT DNA Library Preparation Guide from Illumina (Clooney et al., 2016) and stored at 20°C until further processing. The libraries were assessed using the Agilent 2100 Bioanalyzer system, quantified with the Qubit High Sensitivity DNA kit, and sequenced on the Illumina NextSeq sequencing platform using a v2 NextSeq 500/550 high-output reagent kit (300 cycles) in accordance with standard Illumina sequencing protocols. Sequencing was performed at the Teagasc Sequencing Centre (Moorepark, Cork, Ireland) (Figure S1).

### Bioinformatic analysis

All metagenomic processing was run using the Teagasc high performance computing cluster. Raw paired-end FASTQ files containing the metagenomic shotgun sequences were trimmed using Trimgalore v0.6.1 (Krueger, 2015) to remove adapter content and low quality reads. Reads were queried against a reference bovine genome using Bowtie2 v2.4.4 (Langmead and Salzberg, 2012) to identify contaminated DNA. Taxonomic analysis of the kefir metagenomic data was performed by Kraken 2^25^ against a custom Kraken 2 database. The relative abundance of each species was calculated using Bracken.^46^ The predicted function of the metagenomic sequence data was generated using HUMAnN 3.^47^ Melonnpan v. 0.99^30^ was used to predict the metabolites produced from each kefir microbiome. The top 10 most abundant predicted metabolites detected in ≥10% of samples were then selected for downstream analysis. Metagenomic assembly was performed using MEGAHIT.^48^ MetaWRAP^55^ was used for genome binning, with default settings. CheckM^49^ was implemented to check the quality of metagenome-assembled genomes (MAGs). Lower-quality MAGs, i.e., <80% completeness and/or >5% contamination, were removed from downstream analysis. GTDB-Tk^50^ was used to assign taxonomy to the MAGs and to identify potentially new species. dRep^51^ was used to cluster MAGs representing putative new species into primary and secondary clusters on the basis of their relative similarities. MAGs considered in this study were annotated with DRAM (Distilled and Refined Annotation of Metabolism)^36^ using default parameters.

Strain-level assessment was performed using both PanPhlAn 3^52^ and StrainPhlAn 3.^28^ Phylograms of the StrainPhlAn 3 predicted strains were constructed from multiple sequence alignment (MSA) files of reconstructed markers, as documented in Asnicar et al., 2020. Metacache was used to determine the prevalence and relative abundance of sub-species.^53^

VirSorter2^54^ was used to detect prophage in MAGs and contigs. A cut-off score of 0.5 (default) was selected for maximal sensitivity, and prophage less than 1,500 bp in length were discarded. VirSorter2 detections were then quality inspected using checkV to remove potential host genes bordering predicted viral sequences.^56^ VirSorter2 was again applied on trimmed viral sequences that passed the quality inspection of checkV. Viral sequences obtained through the combined outputs of VirSorter2 and checkV were annotated with the viral mode of DRAM.^36^ For all tools applied (see above), default parameters were used unless specified otherwise. The downstream analysis of data generated by each tool applied was completed in R-4.0.2.^57^

### Quantification and statistical analysis

All statistical analysis was performed in R v4.1.2 implemented through R studio. Statistical analysis of compositional and metabolic data was carried out with vegan v2.6.2,^58^ to compute alpha and beta diversity values. The stats v4.1.2 kruskal.test function was used to perform the Kruskal-Wallis rank sum test, to identify significant differences in alpha diversity values. The adonis function in the vegan package,^58^ was used for permutational analysis of variance (PERMANOVA). PERMDISP^59^ was used to test the null hypothesis of no difference between groups dispersion using vegan v2.6.2. The linear discriminant analysis (LDA) effect size (LEfSe)^60^ was used to identify differentially abundant species and metabolic pathways. Genetic distances between strains were calculated through the dist.DNA function in the package ape v 5.6.2,^61^ using the p-distance, Kimura's 2-parameter evolutionary models and the Jaccard coefficient for PanPhlAn 3 predicted strains. The genetic distances of strains were initially assessed by the Hopkins statistic using the get_clust_tendency function in the package factoextra v1.0.7.^62^ The fviz_nbclust function included in factoextra v1.0.7 was used to predict the optimal number of clusters using the average silhouette method. Cluster validation was performed using the Silhouette coefficient through the fviz_silhouette function in factoextra v1.0.7. Correlation between genetic distances of PanPhlAn 3 and StrainPhlAn 3 predicted strains was calculated using the Kendall correlation method^63^ through the ggscatter function included in ggpubr v0.4.0. Clustering analysis of StrainPhlAn 3 predicted strains was performed using the PAM function of the cluster package v2.1.3 and hierarchical clustering was performed on PanPhlAn 3 predicted strains using the Ward criterion (ward.D) through the stats v4.1.2 hclust function. The species *Propionibacterium freudenreichii* was excluded for clustering due to low sample size. To evaluate if subclusters differed statistically in their gene content, the Mann-Whitney-Wilcoxon test or the Kruskal-Wallis H test was performed. The Mann-Whitney-Wilcoxon tests were performed using the stats v4.1.2 wilcox.test function, the Kruskal-Wallis H tests was performed using the stats v4.1.2 kruskal.test function. The resulting p-values from both functions were corrected for multiple comparisons, using the Bonferroni adjustment method through the stats v4.1.2 p.adjust function.

#### Data visualisation

The mapBubbles function in the package rworldmap v1.3.6,^64^ was used to produce a worldmap of the kefir grains analysed (Figure S1).The experimental design figure was produced using BioRender^65^ (Figure S2). Ordination plots, correlation scatter plots, bar charts and Venn diagrams were produced using ggplot2 v3.3.6.^66^ Heat maps were generated using both ggplot2 and pheatmap v1.0.12.^67^ ggtree v3.2.1^68^ was used to visualise phylogenetic trees from the StrainPhlAn 3 and GTDB-Tk results. The taxize v0.9.100 package^69^ was used to construct a cladogram from the custom Kraken 2 outputs. The resulting cladogram was visualised using the ggtree v3.2.1 package.

## Data Availability

• Sequence data has been deposited in the European Nucleotide Archive (ENA) under the project accession number PRJEB65292 • Computer code used in data analysis is available from the corresponding author upon reasonable request. • Experimental data associated with this study are available in supplemental information.

## Supplemental information

Document S1. Figures S1–S29

Table S1. Metagenomic sequencing details of unfermented milk and milk kefir metagenomes, related to STAR Methods Details include sample id, timepoint, metagenome size [Mbp], number of reads, median read length [bp] of each metagenome generated by this study.

Table S2. Details of reference material downloaded from NCBI to construct the a customized kefir specific database for use in taxonomic profiling, related to STAR Methods Details include taxonomic IDs, species name and the associated genus and superkingdom and lastly the availability of a reference genome at the time of downloading.

Table S3. Species level compositional profile of all 256 kefir metagenomes, as determined using Kraken2, related to Figure 1

Table S4. The taxonomy of the 613 high-quality prokaryotic MAGs, as determined using GTDB-TK, related to Figure 3 Details include bin name, species and genus assignment, % completeness, contamination and strain heterogeneity, FastANI values with the closest known reference genome and novelty. Novelty assesses if the MAGs group into a known species level bin or into a novel species level bin.

Table S5. Details of StrainPhlAn 3 analysis of kefir metagenomic samples, related to Figure 5 Details include outputs of multi-locus sequence alignments for each of the species considered, number of strains and marker genes detected.

Table S6. Pathways detected across kefir metagenomes as determined using HUMAnN 3.0, related to Figure 7 Details include pathway name, code higher classification (MetaCyc) and core pathway identification.

## References

[1] Chin-Wen L., Hsiao-Ling C., Liu J.-R. (1999). Identification and characterisation of lactic acid bacteria and yeasts isolated from kefir grains in Taiwan. Aust. J. Dairy Technol..

[2] Bourrie B.C.T., Willing B.P., Cotter P.D. (2016). The Microbiota and Health Promoting Characteristics of the Fermented Beverage Kefir. Front. Microbiol..

[3] Georgalaki M., Zoumpopoulou G., Anastasiou R., Kazou M., Tsakalidou E. (2021). Lactobacillus kefiranofaciens: From Isolation and Taxonomy to Probiotic Properties and Applications. Microorganisms.

[4] Farnworth E.R., Mainville I. (2003). Kefir: a fermented milk product. Handbook of fermented functional foods.

[5] Blasche S., Kim Y., Mars R.A.T., Machado D., Maansson M., Kafkia E., Milanese A., Zeller G., Teusink B., Nielsen J. (2021). Metabolic cooperation and spatiotemporal niche partitioning in a kefir microbial community. Nat. Microbiol..

[6] Walsh A.M., Crispie F., Kilcawley K., O’Sullivan O., O’Sullivan M.G., Claesson M.J., Cotter P.D. (2016). Microbial succession and flavor production in the fermented dairy beverage kefir. mSystems.

[7] Leeuwendaal N.K., Stanton C., O'Toole P.W., Beresford T.P. (2022). Fermented Foods, Health and the Gut Microbiome. Nutrients.

[8] Slattery C., Cotter P.D., O'Toole P.W. (2019). Analysis of Health Benefits Conferred by Lactobacillus Species from Kefir. Nutrients.

[9] Hertzler S.R., Clancy S.M. (2003). Kefir improves lactose digestion and tolerance in adults with lactose maldigestion. J. Am. Diet Assoc..

[10] Rodrigues K.L., Caputo L.R.G., Carvalho J.C.T., Evangelista J., Schneedorf J.M. (2005). Antimicrobial and healing activity of kefir and kefiran extract. Int. J. Antimicrob. Agents.

[11] Lee M.Y., Ahn K.S., Kwon O.K., Kim M.J., Kim M.K., Lee I.Y., Oh S.R., Lee H.K. (2007). Anti-inflammatory and anti-allergic effects of kefir in a mouse asthma model. Immunobiology.

[12] de Moreno de Leblanc A., Matar C., Farnworth E., Perdigón G. (2007). Study of immune cells involved in the antitumor effect of kefir in a murine breast cancer model. J. Dairy Sci..

[13] Liu J.R., Wang S.Y., Chen M.J., Chen H.L., Yueh P.Y., Lin C.W. (2006). Hypocholesterolaemic effects of milk-kefir and soyamilk-kefir in cholesterol-fed hamsters. Br. J. Nutr..

[14] Bourrie B.C., Cotter P.D., Willing B.P. (2018). Traditional kefir reduces weight gain and improves plasma and liver lipid profiles more successfully than a commercial equivalent in a mouse model of obesity. J. Funct.Foods.

[15] Bourrie B.C.T., Ju T., Fouhse J.M., Forgie A.J., Sergi C., Cotter P.D., Willing B.P. (2021). Kefir microbial composition is a deciding factor in the physiological impact of kefir in a mouse model of obesity. Br. J. Nutr..

[16] van de Wouw M., Walsh A.M., Crispie F., van Leuven L., Lyte J.M., Boehme M., Clarke G., Dinan T.G., Cotter P.D., Cryan J.F. (2020). Distinct actions of the fermented beverage kefir on host behaviour, immunity and microbiome gut-brain modules in the mouse. Microbiome.

[17] Duboc P., Mollet B. (2001). Applications of exopolysaccharides in the dairy industry. Int. Dairy J..

[18] Dimitrellou D., Kandylis P., Mallouchos A., Komaitis M., Koutinas A.A., Kourkoutas Y. (2010). Effect of freeze-dried kefir culture on proteolysis in feta-type and whey-cheeses. Food Chem..

[19] Mantzourani I., Plessas S., Saxami G., Alexopoulos A., Galanis A., Bezirtzoglou E. (2014). Study of kefir grains application in sourdough bread regarding rope spoilage caused by Bacillus spp. Food Chem..

[20] Dimidi E., Cox S.R., Rossi M., Whelan K. (2019). Fermented Foods: Definitions and Characteristics, Impact on the Gut Microbiota and Effects on Gastrointestinal Health and Disease. Nutrients.

[21] Bourrie B.C.T., Richard C., Willing B.P. (2020). Kefir in the Prevention and Treatment of Obesity and Metabolic Disorders. Curr. Nutr. Rep..

[22] Mukherjee A., Gómez-Sala B., O'Connor E.M., Kenny J.G., Cotter P.D. (2022). Global Regulatory Frameworks for Fermented Foods: A Review. Front. Nutr..

[23] Nejati F., Capitain C.C., Krause J.L., Kang G.-U., Riedel R., Chang H.-D., Kurreck J., Junne S., Weller P., Neubauer P. (2022). Traditional Grain-Based vs. Commercial Milk Kefirs, How Different Are They?. Appl. Sci..

[24] Imchen M., Vennapu R.K., Kumavath R., Barh D., Soares S., Tiwari S., Azevedo V. (2020). Pan-genomics: Applications, Challenges, and Future Prospects.

[25] Wood D.E., Lu J., Langmead B. (2019). Improved metagenomic analysis with Kraken 2. Genome Biol..

[26] Beghini F., McIver L.J., Blanco-Míguez A., Dubois L., Asnicar F., Maharjan S., Mailyan A., Manghi P., Scholz M., Thomas A.M. (2021). Integrating taxonomic, functional, and strain-level profiling of diverse microbial communities with bioBakery 3. Elife.

[27] Zolfo M., Pinto F., Asnicar F., Manghi P., Tett A., Bushman F.D., Segata N. (2019). Detecting contamination in viromes using ViromeQC. Nat. Biotechnol..

[28] Truong D.T., Tett A., Pasolli E., Huttenhower C., Segata N. (2017). Microbial strain-level population structure and genetic diversity from metagenomes. Genome Res..

[29] Caspi R., Billington R., Keseler I.M., Kothari A., Krummenacker M., Midford P.E., Ong W.K., Paley S., Subhraveti P., Karp P.D. (2020). The MetaCyc database of metabolic pathways and enzymes - a 2019 update. Nucleic Acids Res..

[30] Mallick H., Franzosa E.A., McLver L.J., Banerjee S., Sirota-Madi A., Kostic A.D., Clish C.B., Vlamakis H., Xavier R.J., Huttenhower C. (2019). Predictive metabolomic profiling of microbial communities using amplicon or metagenomic sequences. Nat. Commun..

[31] Wu Q., Tun H.M., Law Y.-S., Khafipour E., Shah N.P. (2017). Common Distribution of gad Operon in Lactobacillus brevis and its GadA Contributes to Efficient GABA Synthesis toward Cytosolic Near-Neutral pH. Front. Microbiol..

[32] Diez-Gutiérrez L., San Vicente L., R Barrón L.J., Villarán M.d.C., Chávarri M., Chávarri M. (2020). Gamma-aminobutyric acid and probiotics: Multiple health benefits and their future in the global functional food and nutraceuticals market. J. Funct.Foods.

[33] Li T.T., Tian W.L., Gu C.T. (2019). Elevation of Lactococcus lactis subsp. cremoris to the species level as Lactococcus cremoris sp. nov. and transfer of Lactococcus lactis subsp. tructae to Lactococcus cremoris as Lactococcus cremoris subsp. tructae comb. nov. Int. J. Syst. Evol. Microbiol..

[34] Richter M., Rosselló-Móra R. (2009). Shifting the genomic gold standard for the prokaryotic species definition. Proc. Natl. Acad. Sci. USA..

[35] International Code of Nomenclature of Prokaryotes (2019). Int. J. Syst. Evol. Microbiol..

[36] Shaffer M., Borton M.A., McGivern B.B., Zayed A.A., La Rosa S.L., Solden L.M., Liu P., Narrowe A.B., Rodríguez-Ramos J., Bolduc B. (2020). DRAM for distilling microbial metabolism to automate the curation of microbiome function. Nucleic Acids Res..

[37] Warwick-Dugdale J., Buchholz H.H., Allen M.J., Temperton B. (2019). Host-hijacking and planktonic piracy: how phages command the microbial high seas. Virol. J..

[38] Muda M., Rao N.N., Torriani A. (1992). Role of PhoU in phosphate transport and alkaline phosphatase regulation. J. Bacteriol..

[39] Wels M., Siezen R., Van Hijum S., Kelly W.J., Bachmann H. (2019). Comparative genome analysis of Lactococcus lactis indicates niche adaptation and resolves genotype/phenotype disparity. Front. Microbiol..

[40] Farag M.A., Jomaa S.A., El-Wahed A.A., El-Seedi A.H.R. (2020). The Many Faces of Kefir Fermented Dairy Products: Quality Characteristics, Flavour Chemistry, Nutritional Value, Health Benefits, and Safety. Nutrients.

[41] Cryan J.F., O'Leary O.F. (2010). A glutamate pathway to faster-acting antidepressants?. Science.

[42] Guangsen T., Xiang L., Jiahu G. (2021). Microbial diversity and volatile metabolites of kefir prepared by different milk types. CyTA - J. Food.

[43] Bratbak G., Egge J.K., Heldal M. (1993). Viral mortality of the marine alga Emiliania huxleyi (Haptophyceae) and termination of algal blooms. Mar. Ecol. Prog. Ser..

[44] CLASEN J.L., ELSER J.J. (2007). The effect of host Chlorella NC64A carbon : phosphorus ratio on the production of Paramecium bursaria Chlorella Virus-1. Freshw. Biol..

[45] Walsh L.H., Coakley M., Walsh A.M., O’Toole P.W., Cotter P.D. (2022). Bioinformatic approaches for studying the microbiome of fermented food. Crit. Rev. Microbiol..

[46] Lu J., Breitwieser F.P., Thielen P., Salzberg S.L. (2017). Bracken: estimating species abundance in metagenomics data. PeerJ Comput. Sci..

[47] Beghini F., McIver L.J., Blanco-Míguez A., Dubois L., Asnicar F., Maharjan S., Mailyan A., Manghi P., Scholz M., Thomas A.M. (2021). Integrating taxonomic, functional, and strain-level profiling of diverse microbial communities with bioBakery 3. Elife.

[48] Li D., Liu C.M., Luo R., Sadakane K., Lam T.W. (2015). MEGAHIT: an ultra-fast single-node solution for large and complex metagenomics assembly via succinct de Bruijn graph. Bioinformatics.

[49] Parks D.H., Imelfort M., Skennerton C.T., Hugenholtz P., Tyson G.W. (2015). CheckM: assessing the quality of microbial genomes recovered from isolates, single cells, and metagenomes. Genome Res..

[50] Chaumeil P.-A., Mussig A.J., Hugenholtz P., Parks D.H. (2019). GTDB-Tk: a toolkit to classify genomes with the Genome Taxonomy Database. Bioinformatics.

[51] Olm M.R., Brown C.T., Brooks B., Banfield J.F. (2017). dRep: a tool for fast and accurate genomic comparisons that enables improved genome recovery from metagenomes through de-replication. ISME J..

[52] Asnicar F., Thomas A.M., Beghini F., Mengoni C., Manara S., Manghi P., Zhu Q., Bolzan M., Cumbo F., May U. (2020). Precise phylogenetic analysis of microbial isolates and genomes from metagenomes using PhyloPhlAn 3.0. Nat. Commun..

[53] Müller A., Hundt C., Hildebrandt A., Hankeln T., Schmidt B. (2017). MetaCache: context-aware classification of metagenomic reads using minhashing. Bioinformatics.

[54] Guo J., Bolduc B., Zayed A.A., Varsani A., Dominguez-Huerta G., Delmont T.O., Pratama A.A., Gazitúa M.C., Vik D., Sullivan M.B., Roux S. (2021). VirSorter2: a multi-classifier, expert-guided approach to detect diverse DNA and RNA viruses. Microbiome.

[55] Uritskiy G.V., DiRuggiero J., Taylor J. (2018). MetaWRAP-a flexible pipeline for genome-resolved metagenomic data analysis. Microbiome.

[56] Nayfach S., Camargo A.P., Schulz F., Eloe-Fadrosh E., Roux S., Kyrpides N.C. (2021). CheckV assesses the quality and completeness of metagenome-assembled viral genomes. Nat. Biotechnol..

[57] R Core Team (2020).

[58] Oksanen J., Blanchet F.G., Friendly M., Kindt R., Legendre P., McGlinn D., Minchin R.P., O'Hara R.B., Simpson G.L., Solymos P. (2020).

[59] Anderson M.J. (2006). Distance-based tests for homogeneity of multivariate dispersions. Biometrics.

[60] Segata N., Izard J., Waldron L., Gevers D., Miropolsky L., Garrett W.S., Huttenhower C. (2011). Metagenomic biomarker discovery and explanation. Genome Biol..

[61] Paradis E., Schliep K. (2019). ape 5.0: an environment for modern phylogenetics and evolutionary analyses in R. Bioinformatics.

[62] Kassambara A., Mundt F. (2020).

[63] Kassambara A. (2020).

[64] South A., South M.A. (2016). Package ‘rworldmap’. Mapping Global Data.

[65] BioRender (2021).

[66] Wickham H. (2016).

[67] Kolde R. (2019).

[68] Yu G. (2020). Using ggtree to Visualize Data on Tree-Like Structures. Curr. Protoc. Bioinformatics.

[69] Chamberlain S.A., Szöcs E. (2013). taxize: taxonomic search and retrieval in R. F1000Res..

