# Supplementary material for: Analysis of the milk kefir pan-metagenome reveals four community types, core species, and associated metabolic pathways
Source: iScience. 2023 Sep 21;26(10):108004. doi: 10.1016/j.isci.2023.108004 (PMC10568436; doi:10.1016/j.isci.2023.108004)
Supplement: Document S1. Figures S1–S29 [file mmc1.pdf]

iScience, Volume *26*

Supplemental information

Analysis of the milk kefir pan-metagenome reveals four community types, core species, and associated metabolic pathways

Liam H. Walsh, Mairéad Coakley, Aaron M. Walsh, Fiona Crispie, Paul W. O'Toole, and Paul D. Cotter

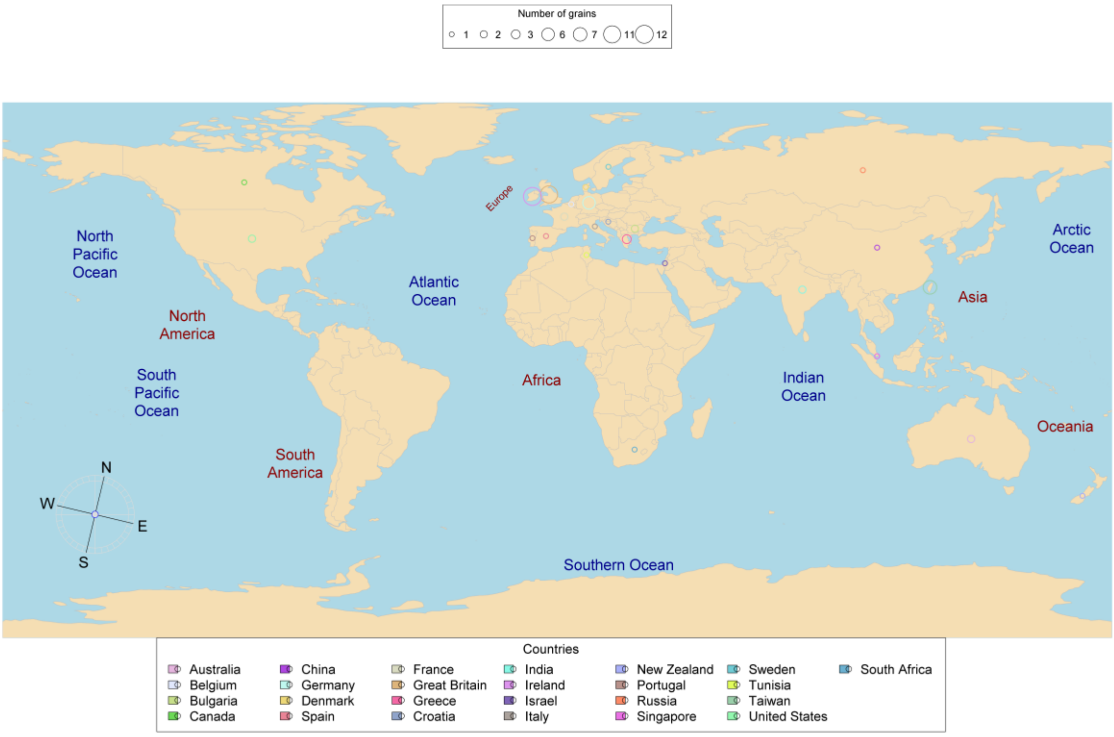

Figure S1. Kefir grain sampling initiative undertaken in this study, Related to Figure 1, Figure 2 and Figure 7. World map showing the countries from which the kefir grains were sourced. The different colour circles represent the country in which the kefir grains originated. The diameter of the circle displays the number of grains sourced from that country.

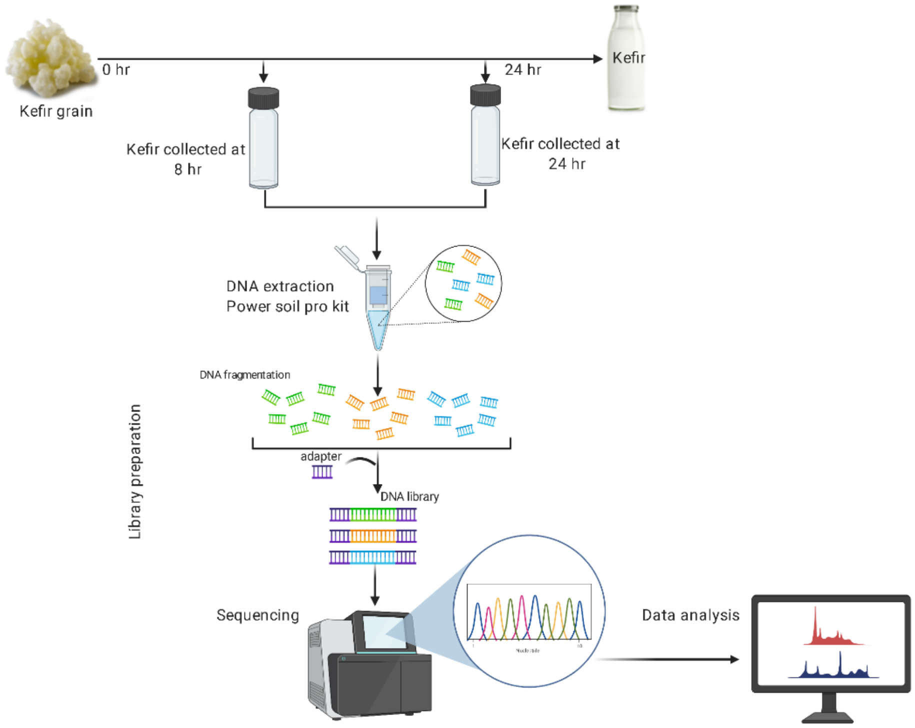

Figure S2. Graphical representation of the experimental design implemented in this study, Related to Figure 1, Figure 2 and Figure 7. Steps involved in the experimental design included kefir fermentations, sample collection, DNA extraction, library preparation, sequencing and bioinformatics analysis.

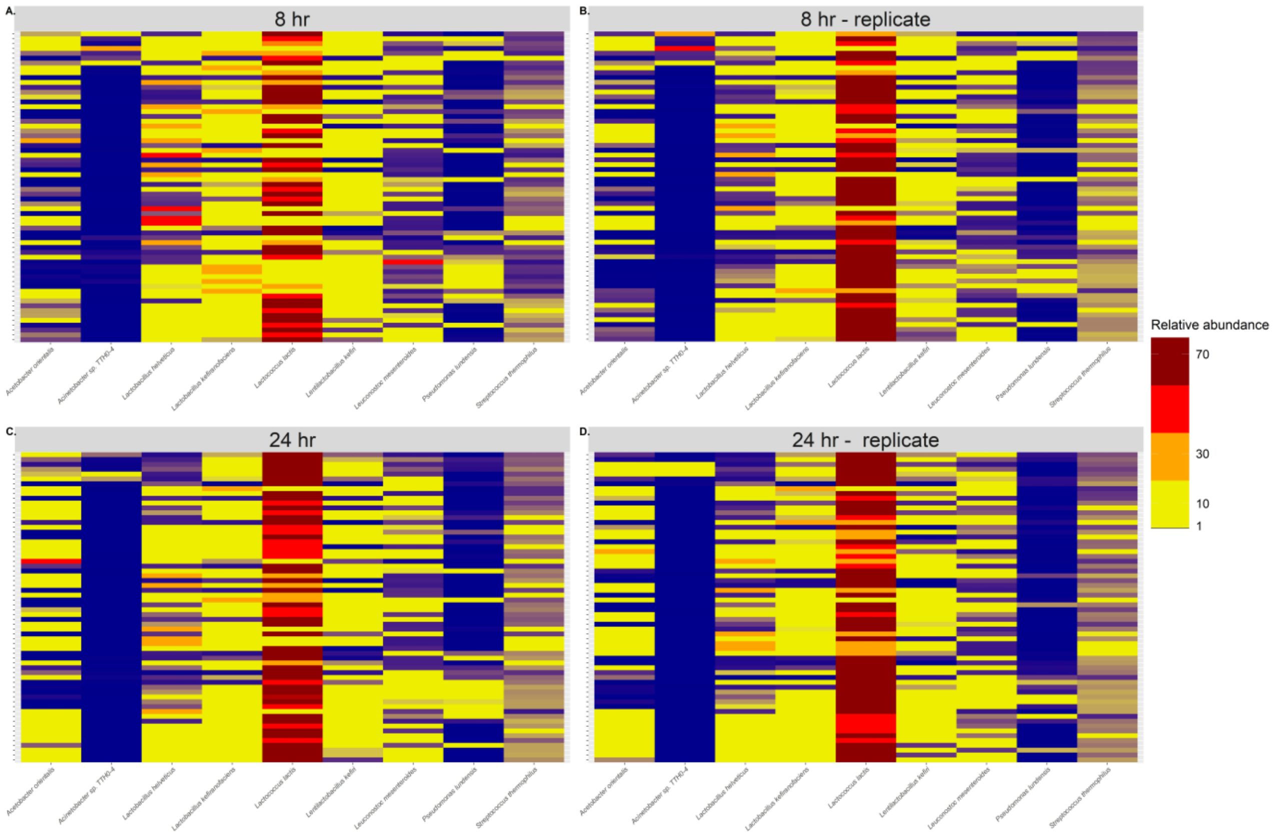

Figure S3. **The microbial community patterns of the milk kefirs with time**, Related to Figure 1. **A.** Species that were detected at ≥10% relative abundance in at least one 8 hr sample. **B.** Species that were detected at ≥10% relative abundance in at least one 8 hr-replicate sample. **C.** Species that were detected at ≥10% relative abundance in at least one 24 hr sample and **D.** Species that were detected at ≥10% relative abundance in at least one 24 hr-replicate sample.

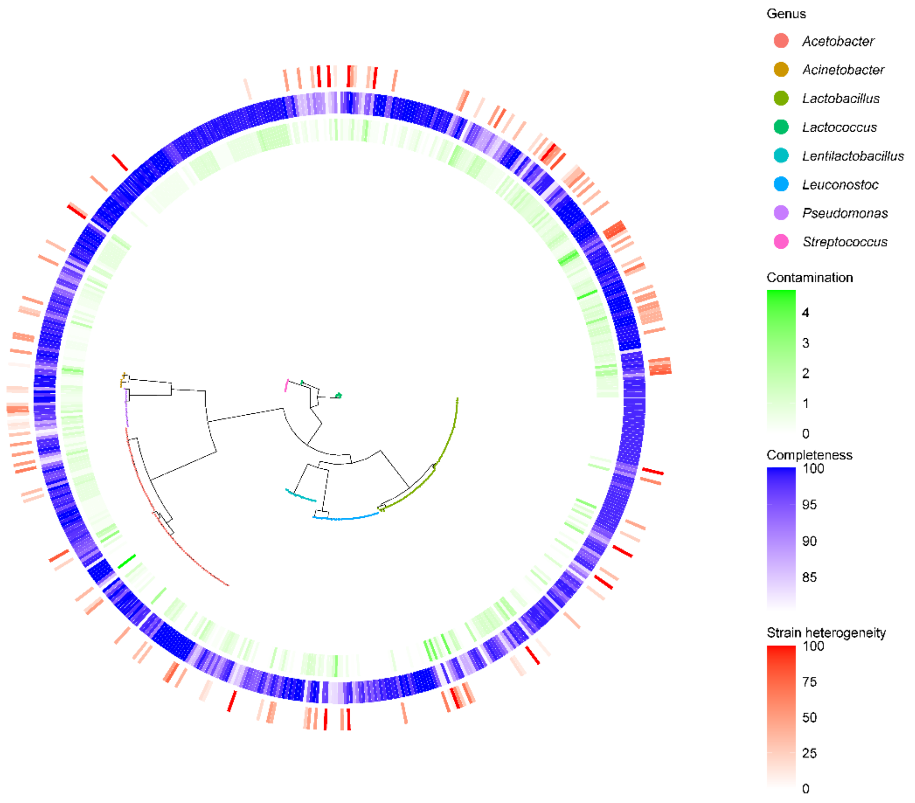

Figure S4. High quality metagenome assembled genomes assembled in this study, Related to Figure 3. Phylogenetic tree of the 613 high quality MAGs assembled from kefir metagenomics datasets, obtained in this study. Outer rings coloured green, blue and red represent % contamination, completion and strain heterogeneity respectively, as assessed using checkM. The coloured tip of the phylogram corresponding to the genus classification of the MAG.

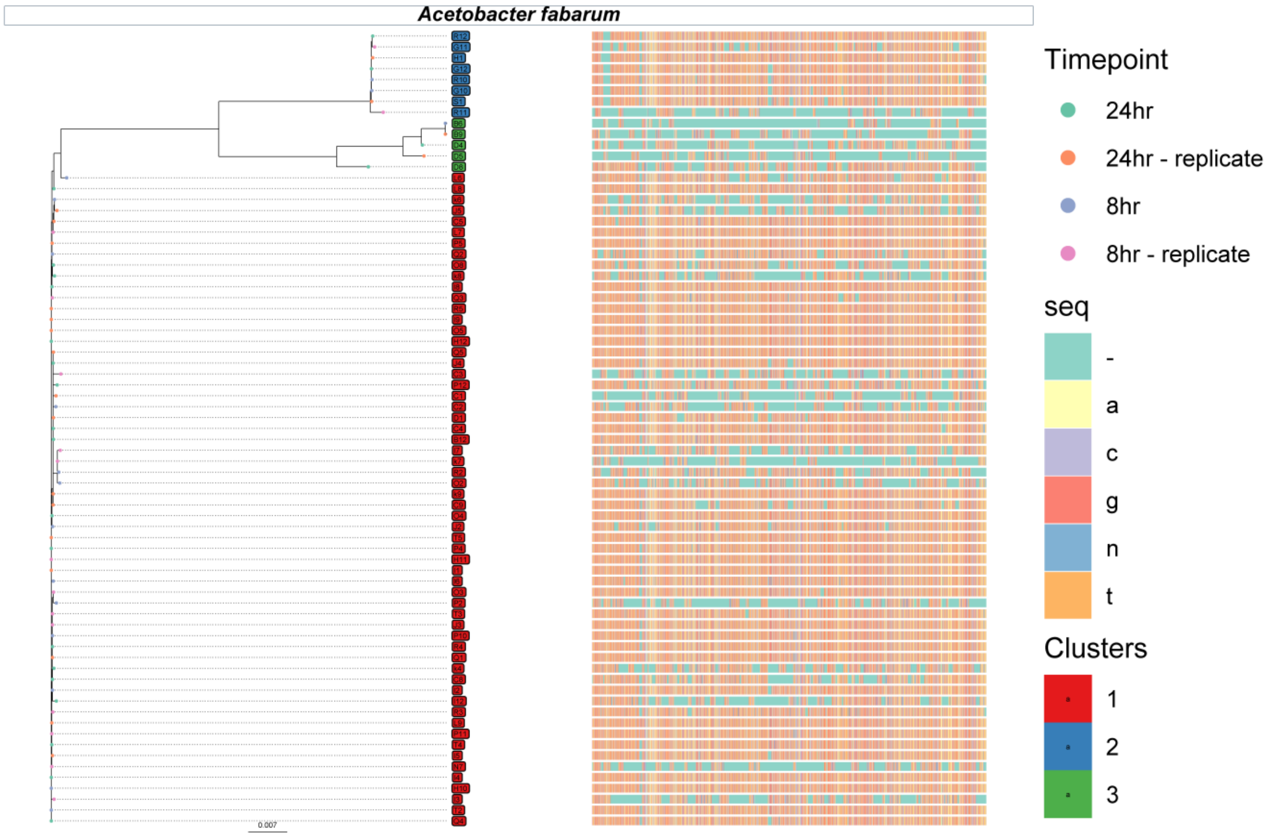

Figure S5. Strains of *A. fabarum* strains detected across kefir metagenomes, Related to Figure 4. Strain level phylograms of *A. fabarum* strains and corresponding multiple sequence alignment (MSA) files generated by StrainPhlAn 3. Coloured tips of the phylogram corresponds to the time point of the kefir metagenome from which the strain was recovered. Colouring of labels represents the strain subcluster by which the strain was grouped.

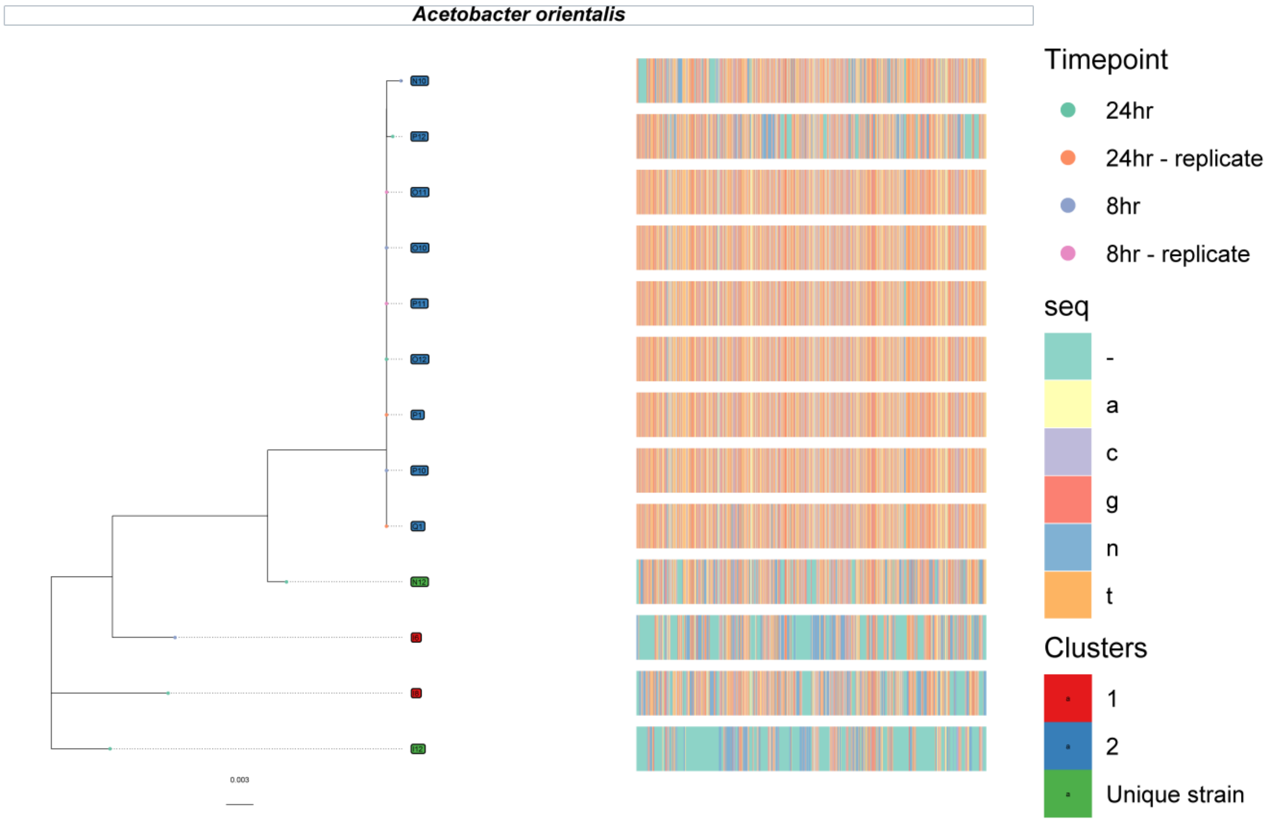

Figure S6. Strains of *A. orientalis* strains detected across kefir metagenomes, Related to Figure 4. Strain level phylograms of *A. orientalis* strains and corresponding multiple sequence alignment (MSA) files generated by StrainPhlAn 3. Coloured tips of the phylogram corresponds to the time point of the kefir metagenome from which the strain was recovered. Colouring of labels represents the strain subcluster by which the strain was grouped.

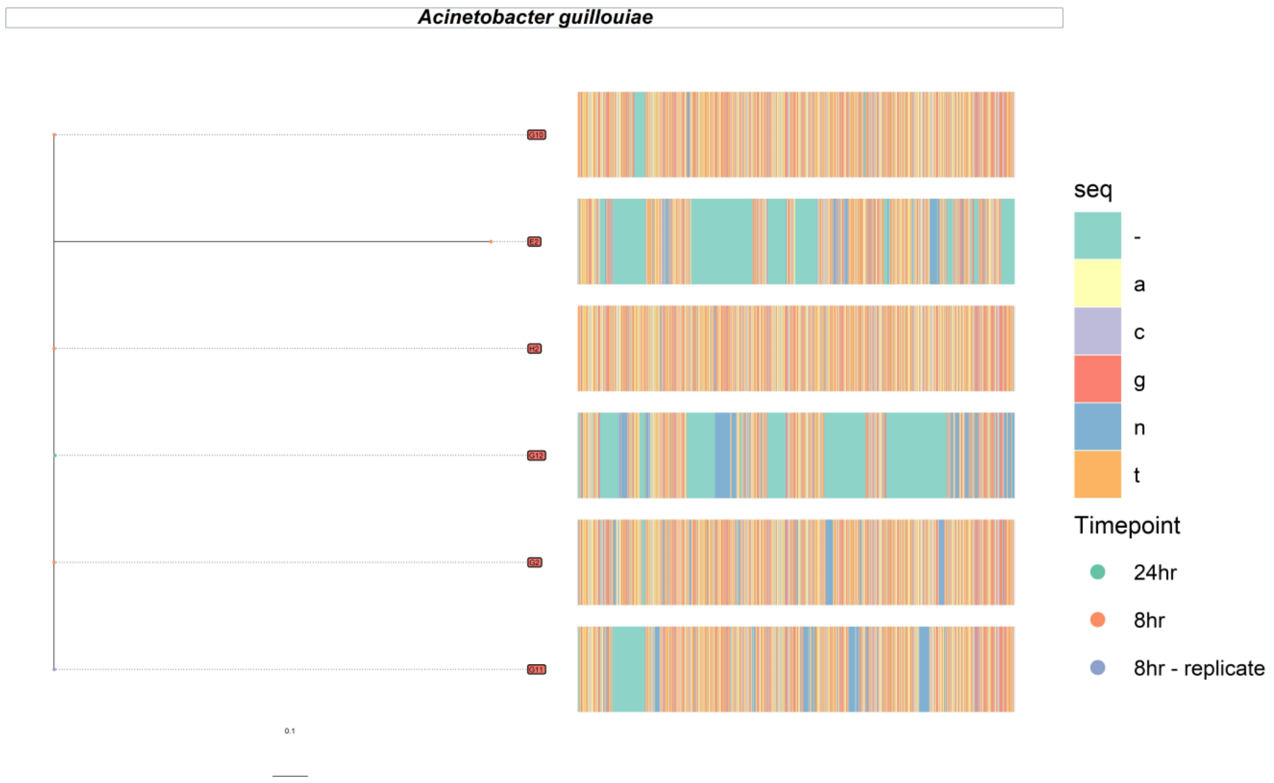

Figure S7. Strains of *Ac. guillouiae* strains detected across kefir metagenomes, Related to Figure 4. Strain level phylograms of *Ac. guillouiae* strains and corresponding multiple sequence alignment (MSA) files generated by StrainPhlAn 3. Coloured tips of the phylogram corresponds to the time point of the kefir metagenome from which the strain was recovered. Colouring of labels represents the strain subcluster by which the strain was grouped.

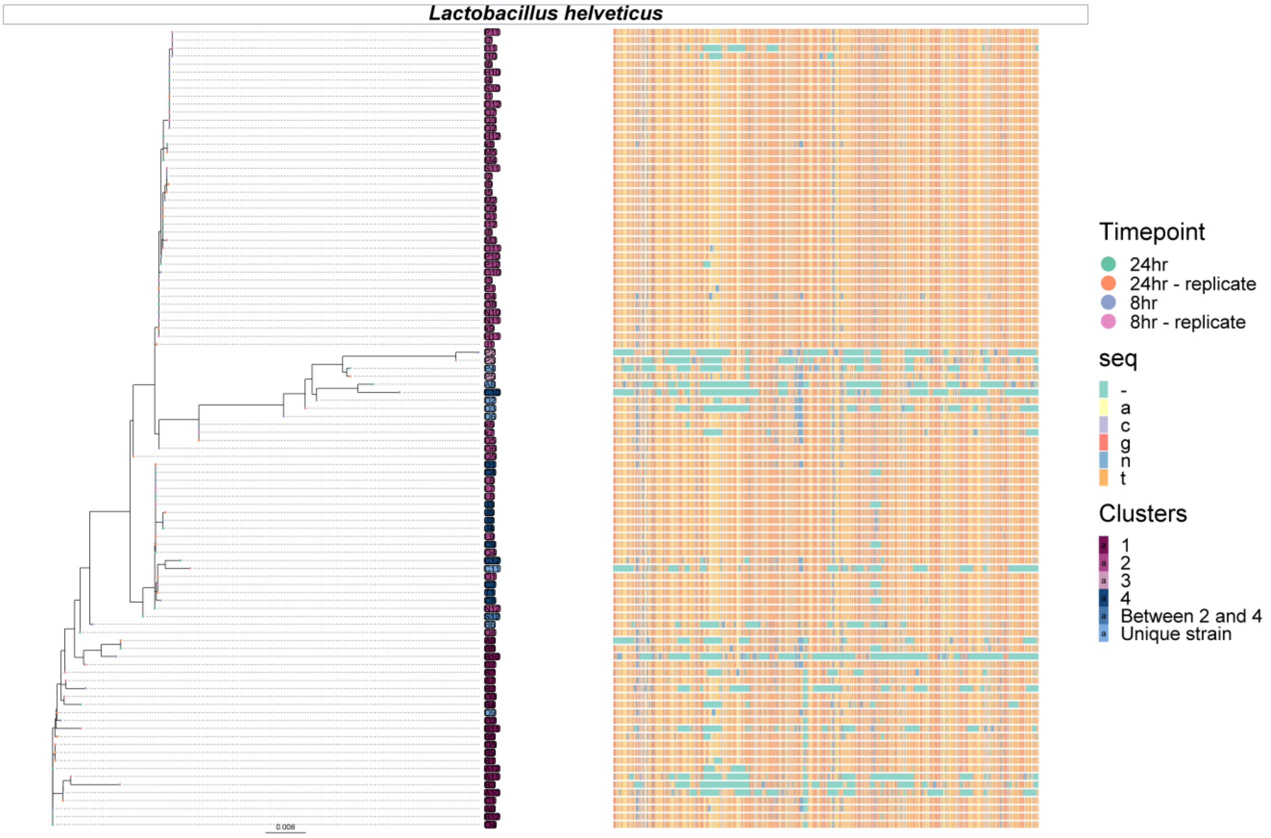

Figure S8. Strains of *L. helveticus* strains detected across kefir metagenomes, Related to Figure 4. Strain level phylograms of *L. helveticus* strains and corresponding multiple sequence alignment (MSA) files generated by StrainPhlAn 3. Coloured tips of the phylogram corresponds to the time point of the kefir metagenome from which the strain was recovered. Colouring of labels represents the strain subcluster by which the strain was grouped.

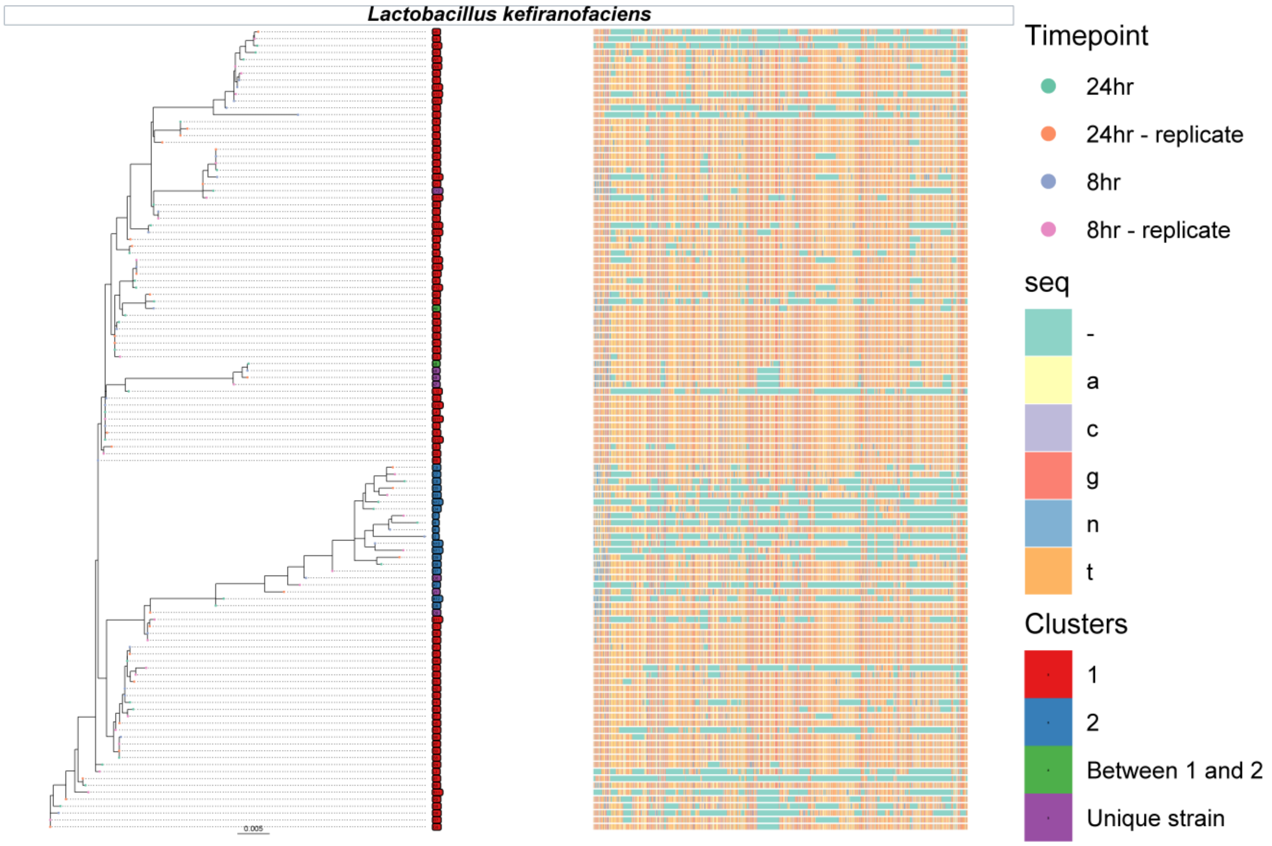

Figure S9. Strains of *L. kefiranofaciens* strains detected across kefir metagenomes, Related to Figure 4. Strain level phylograms of *L. kefiranofaciens* strains and corresponding multiple sequence alignment (MSA) files generated by StrainPhlAn 3. Coloured tips of the phylogram corresponds to the time point of the kefir metagenome from which the strain was recovered. Colouring of labels represents the strain subcluster by which the strain was grouped.

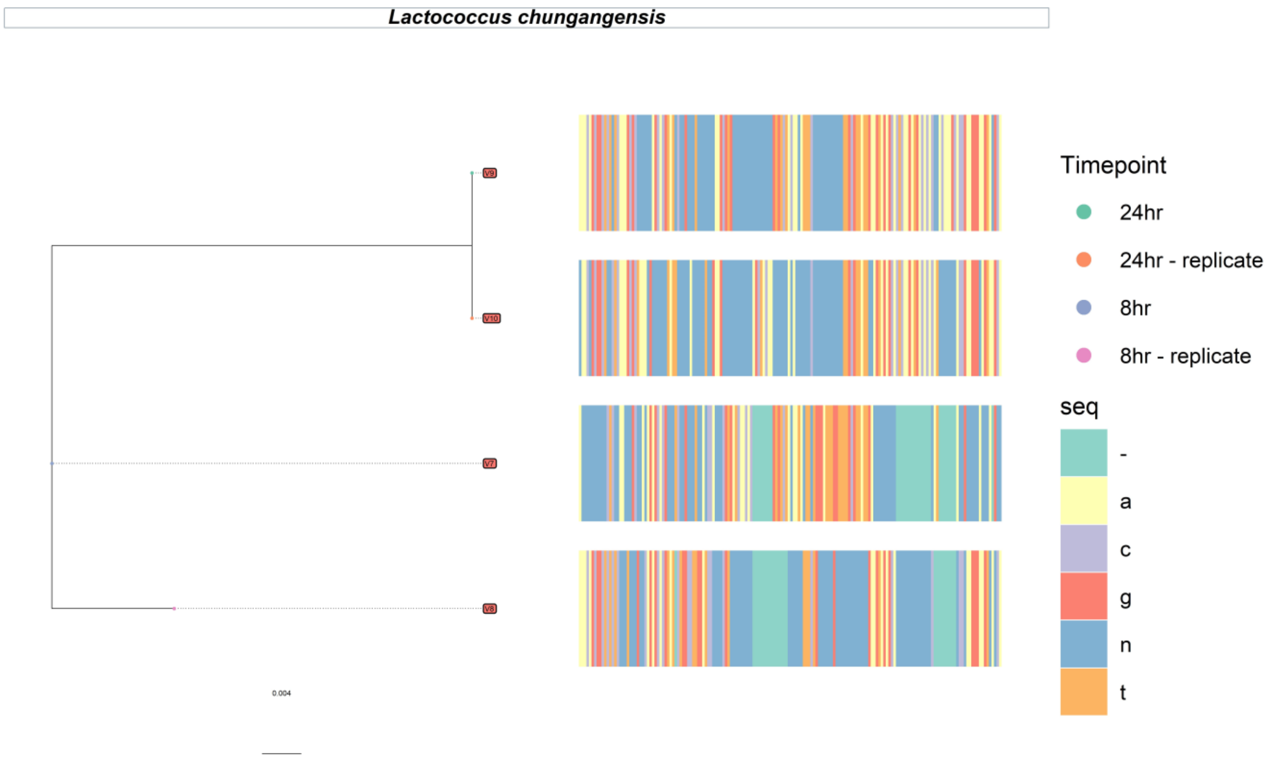

Figure S10. Strains of *Lc. chungangensis* strains detected across kefir metagenomes, Related to Figure 4. Strain level phylograms of *Lc. chungangensis* strains and corresponding multiple sequence alignment (MSA) files generated by StrainPhlAn 3. Coloured tips of the phylogram corresponds to the time point of the kefir metagenome from which the strain was recovered. Colouring of labels represents the strain subcluster by which the strain was grouped.

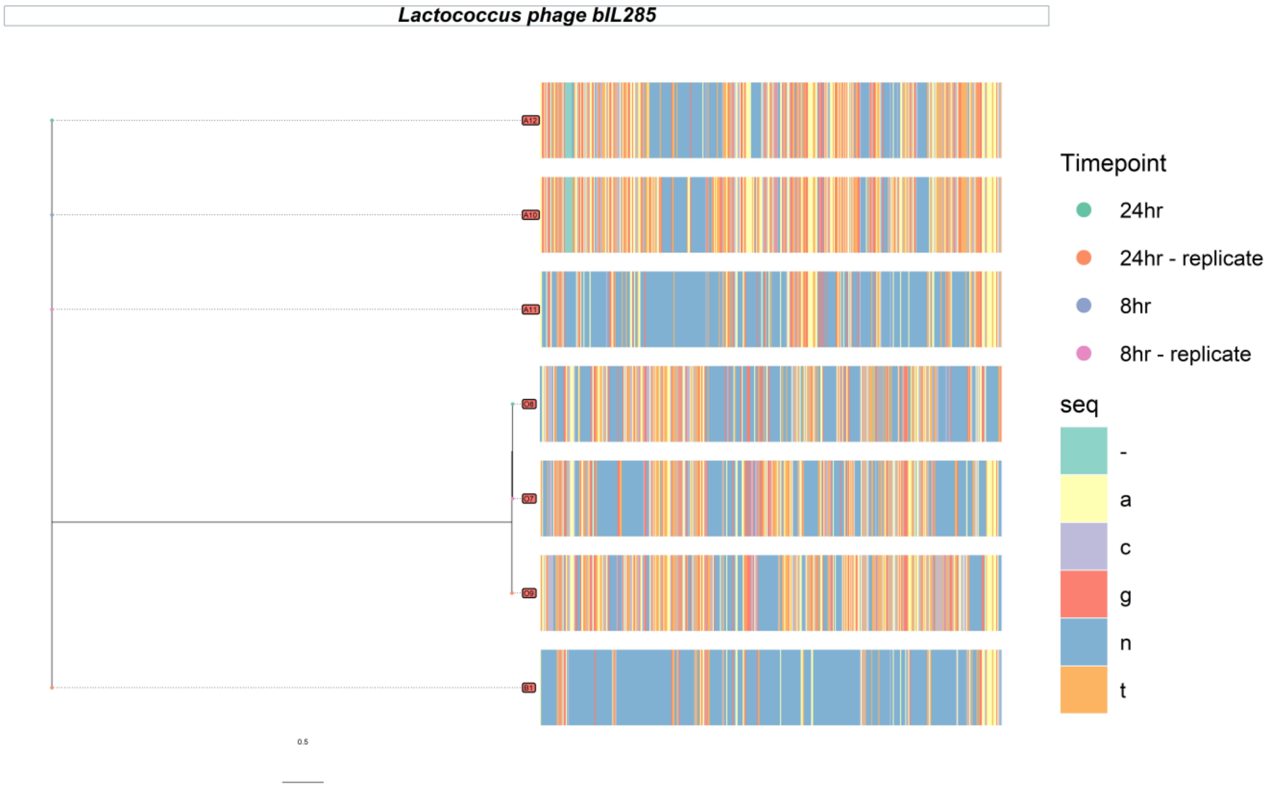

Figure S11. Strains of *Lc. phage bIL285* strains detected across kefir metagenomes, Related to Figure 4. Strain level phylograms of *Lc. phage bIL285* strains and corresponding multiple sequence alignment (MSA) files generated by StrainPhlAn 3. Coloured tips of the phylogram corresponds to the time point of the kefir metagenome from which the strain was recovered. Colouring of labels represents the strain subcluster by which the strain was grouped.

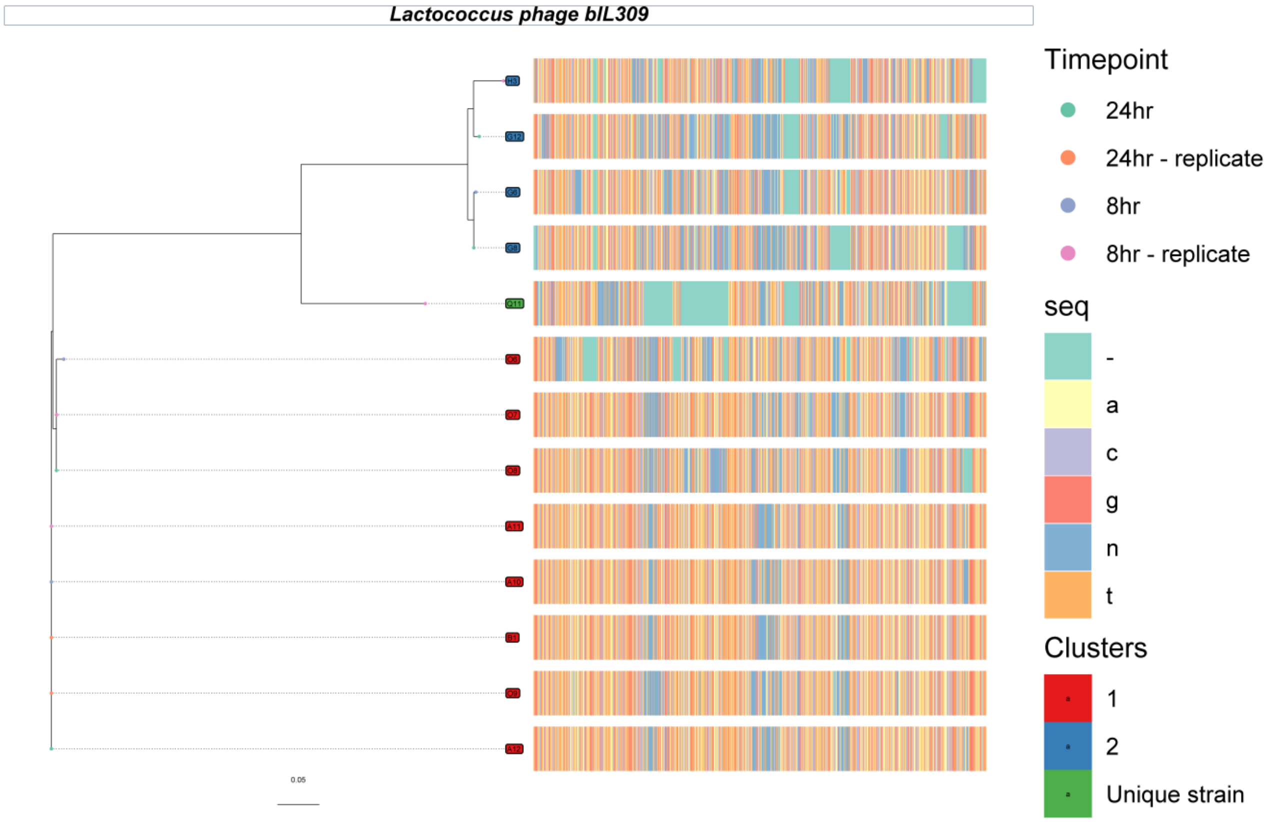

Figure S12. Strains of *Lc. phage bIL309* strains detected across kefir metagenomes, Related to Figure 4. Strain level phylograms of *Lc. phage bIL309* strains and corresponding multiple sequence alignment (MSA) files generated by StrainPhlAn 3. Coloured tips of the phylogram corresponds to the time point of the kefir metagenome from which the strain was recovered. Colouring of labels represents the strain subcluster by which the strain was grouped.

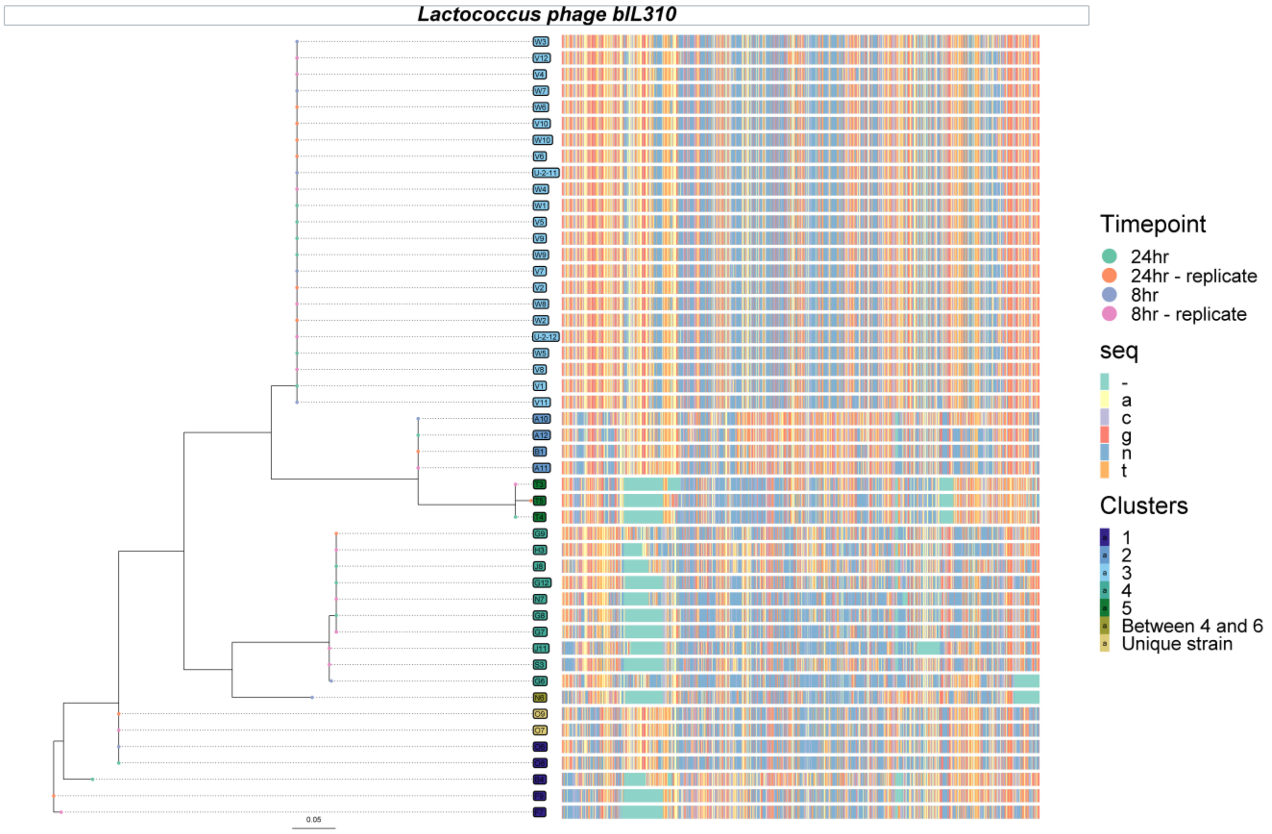

Figure S13. Strains of *Lc. phage bIL310* strains detected across kefir metagenomes, Related to Figure 4. Strain level phylograms of *Lc.* phage *bIL310* strains and corresponding multiple sequence alignment (MSA) files generated by StrainPhlAn 3. Coloured tips of the phylogram corresponds to the time point of the kefir metagenome from which the strain was recovered. Colouring of labels represents the strain subcluster by which the strain was grouped.

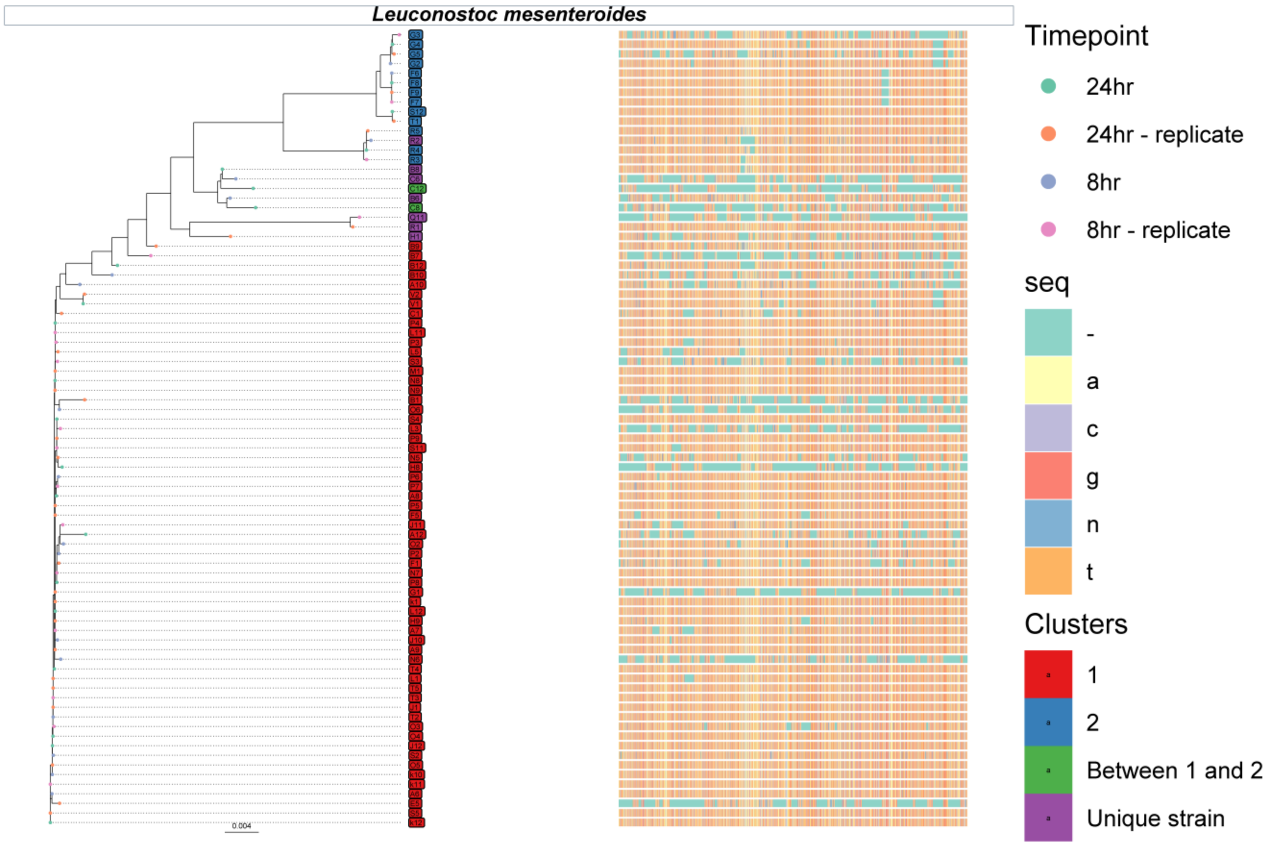

Figure S14. Strains of *Leuc. mesenteroides* strains detected across kefir metagenomes, Related to Figure 4. Strain level phylograms of *Leuc. mesenteroides* strains and corresponding multiple sequence alignment (MSA) files generated by StrainPhlAn 3. Coloured tips of the phylogram corresponds to the time point of the kefir metagenome from which the strain was recovered. Colouring of labels represents the strain subcluster by which the strain was grouped.

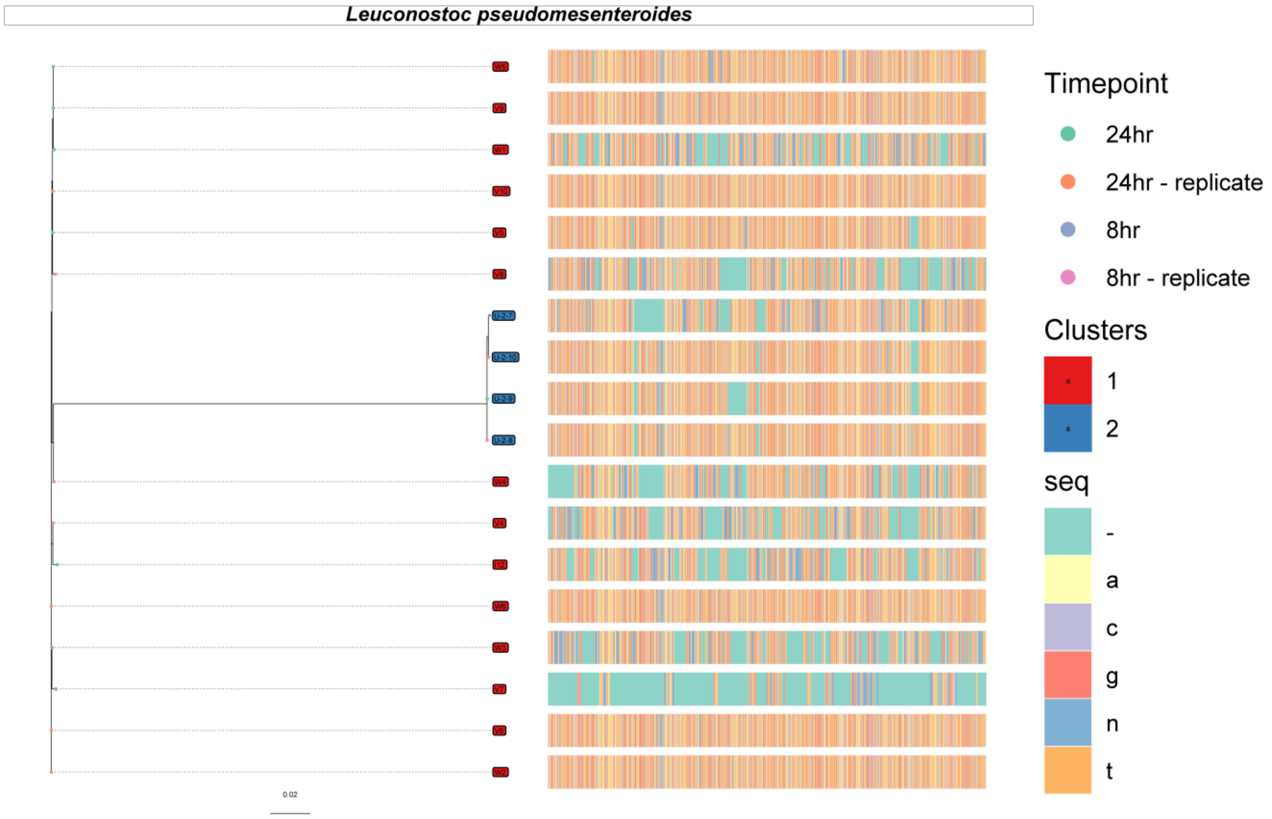

Figure S15. Strains of *Leuc. pseudomesenteroides* strains detected across kefir metagenomes, Related to Figure 4. Strain level phylograms of *Leuc. pseudomesenteroides* strains and corresponding multiple sequence alignment (MSA) files generated by StrainPhlAn 3. Coloured tips of the phylogram corresponds to the time point of the kefir metagenome from which the strain was recovered. Colouring of labels represents the strain subcluster by which the strain was grouped.

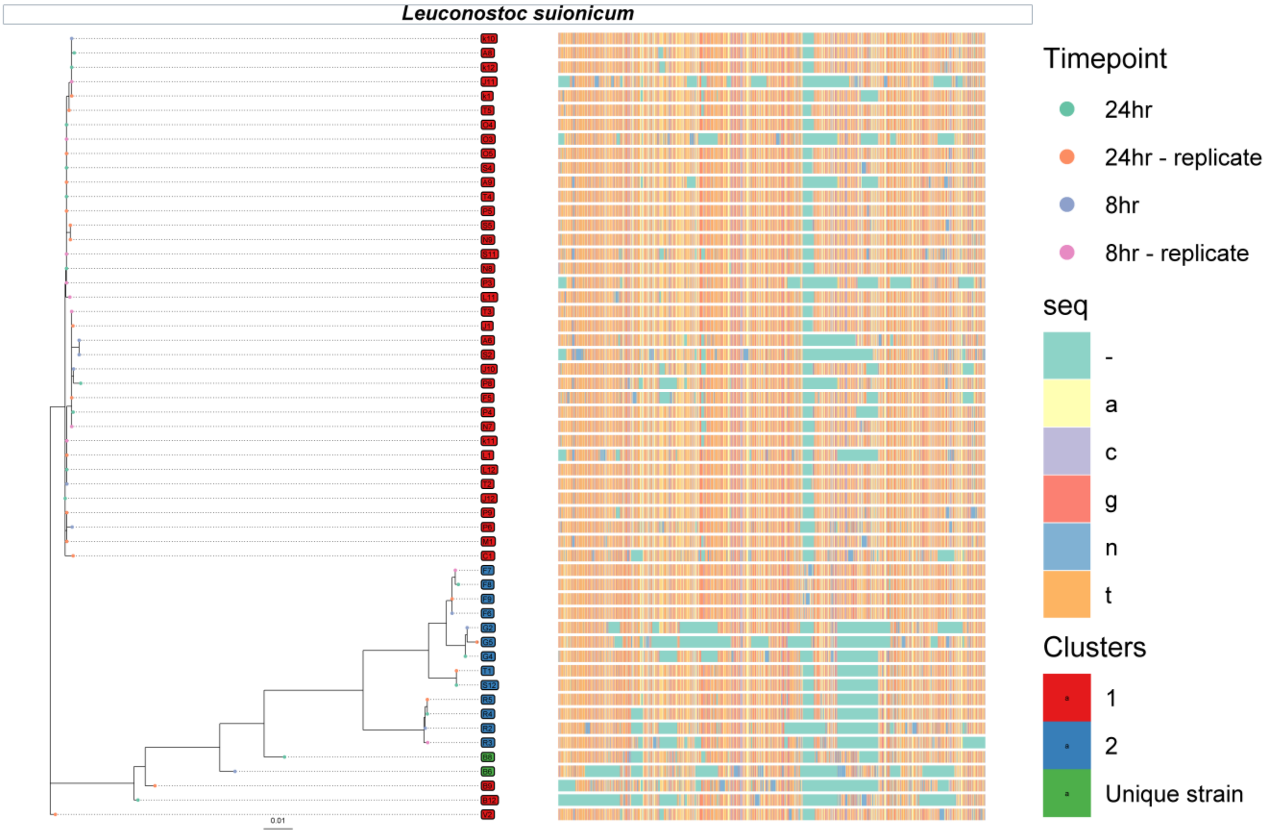

Figure S16. Strains of *Leuc. suionicum* strains detected across kefir metagenomes, Related to Figure 4. Strain level phylograms of *Leuc. suionicum* strains and corresponding multiple sequence alignment (MSA) files generated by StrainPhlAn 3. Coloured tips of the phylogram corresponds to the time point of the kefir metagenome from which the strain was recovered. Colouring of labels represents the strain subcluster by which the strain was grouped.

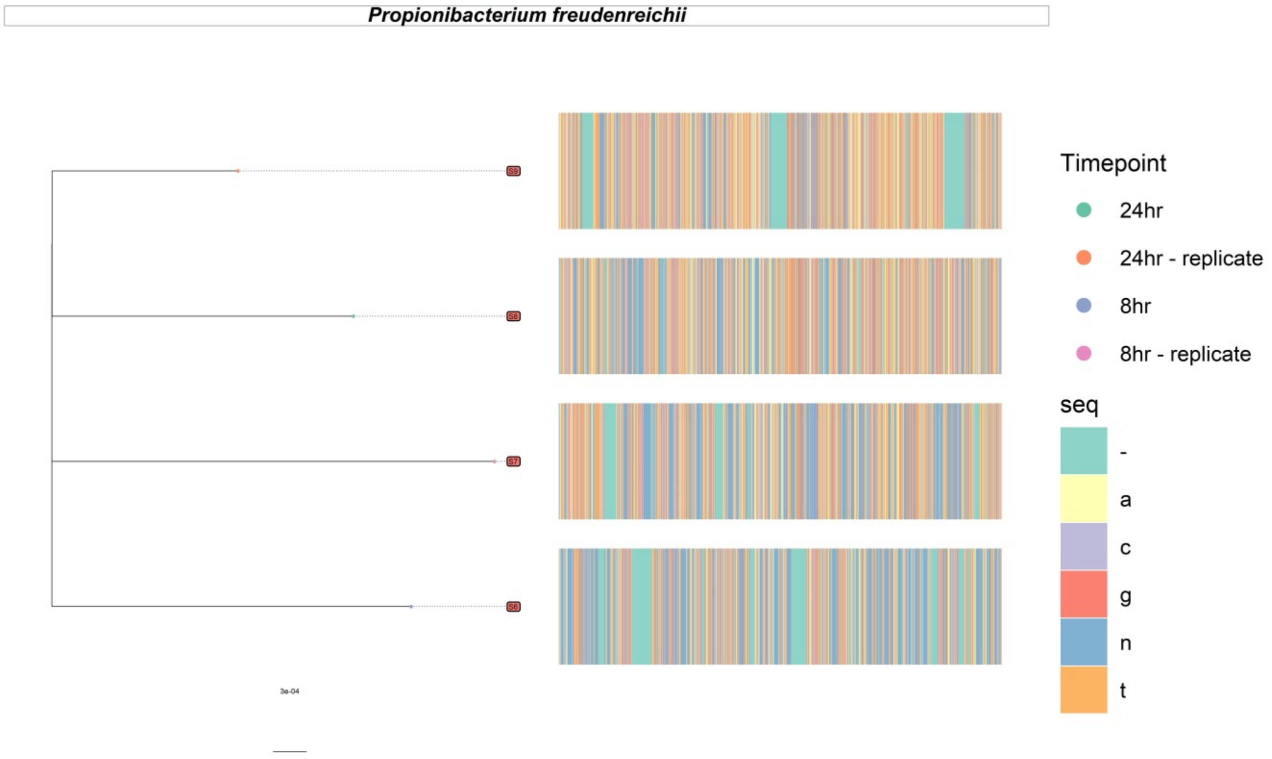

Figure S17. Strains of *Pr. freudenreichii* strains detected across kefir metagenomes, Related to Figure 4. Strain level phylograms of *Pr. freudenreichii* strains and corresponding multiple sequence alignment (MSA) files generated by StrainPhlAn 3. Coloured tips of the phylogram corresponds to the time point of the kefir metagenome from which the strain was recovered. Colouring of labels represents the strain subcluster by which the strain was grouped.

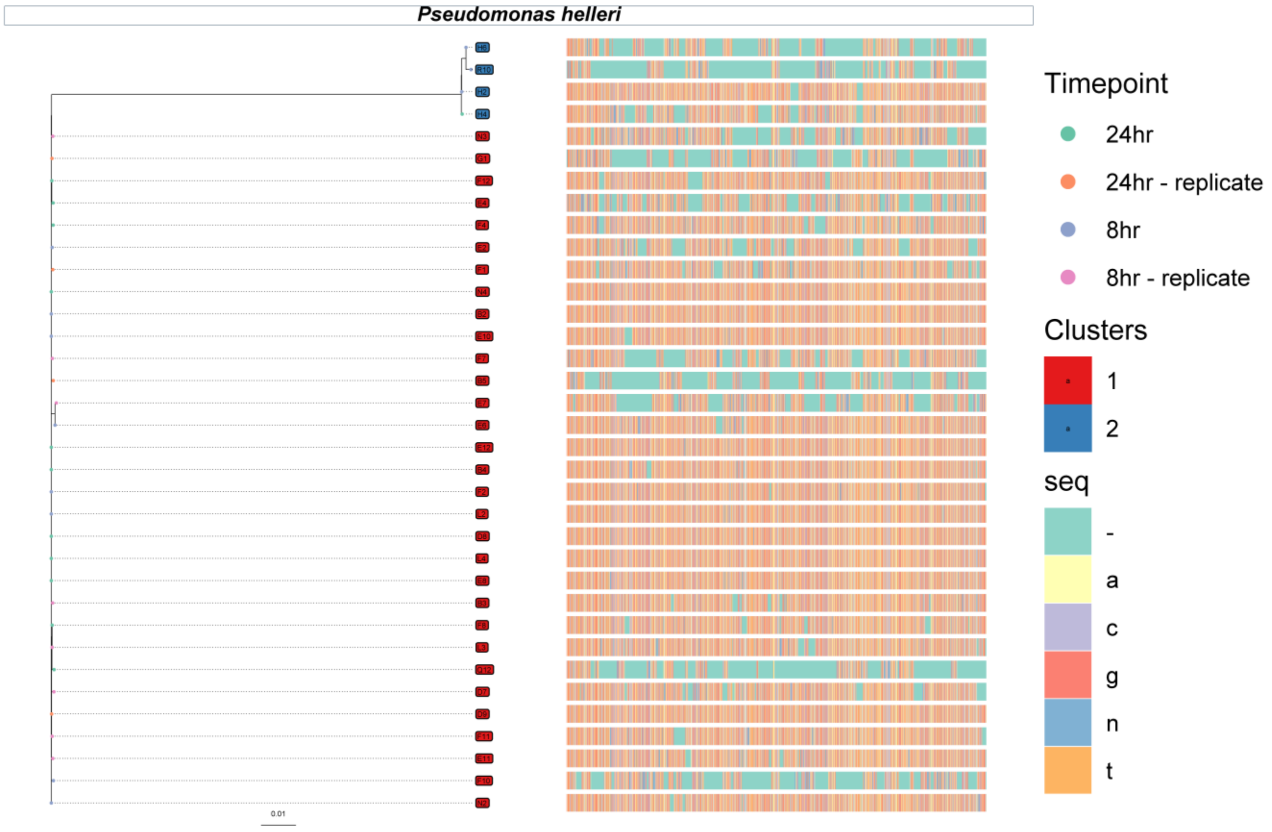

Figure S18. Strains of *P. helleri* strains detected across kefir metagenomes, Related to Figure 4. Strain level phylograms of *P. helleri* strains and corresponding multiple sequence alignment (MSA) files generated by StrainPhlAn 3. Coloured tips of the phylogram corresponds to the time point of the kefir metagenome from which the strain was recovered. Colouring of labels represents the strain subcluster by which the strain was grouped.

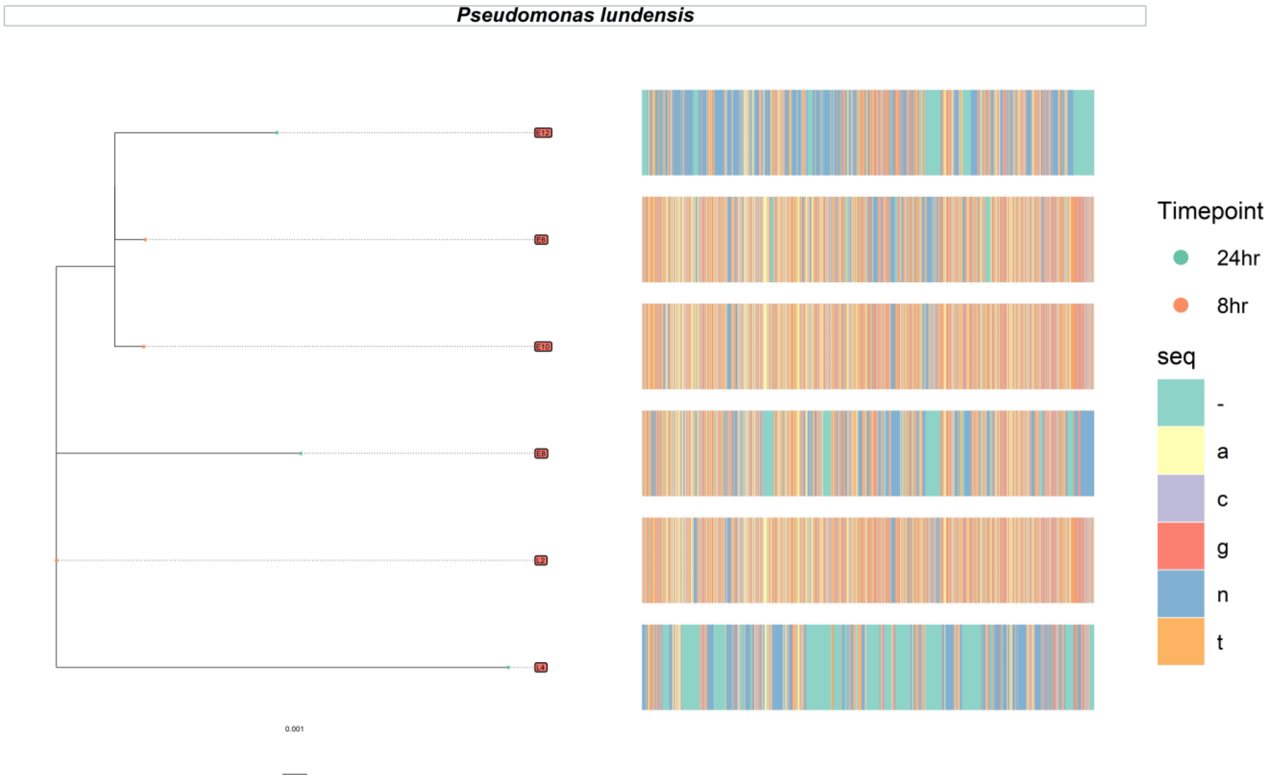

Figure S19. Strains of *P. lundensis* strains detected across kefir metagenomes, Related to Figure 4. Strain level phylograms of *P. lundensis* strains and corresponding multiple sequence alignment (MSA) files generated by StrainPhlAn 3. Coloured tips of the phylogram corresponds to the time point of the kefir metagenome from which the strain was recovered. Colouring of labels represents the strain subcluster by which the strain was grouped.

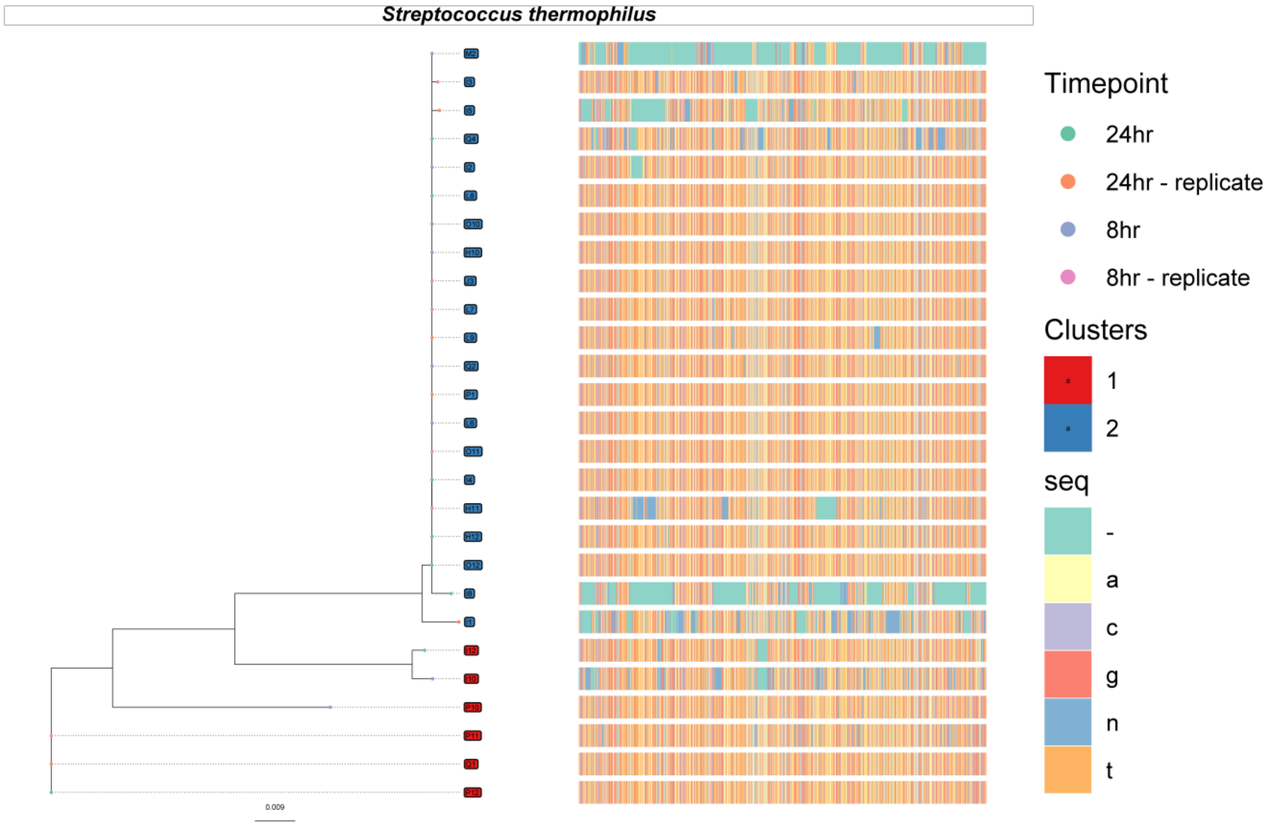

Figure S20. Strains of *S. thermophilus* strains detected across kefir metagenomes, Related to Figure 4. Strain level phylograms of *S. thermophilus* strains and corresponding multiple sequence alignment (MSA) files generated by StrainPhlAn 3. Coloured tips of the phylogram corresponds to the time point of the kefir metagenome from which the strain was recovered. Colouring of labels represents the strain subcluster by which the strain was grouped.

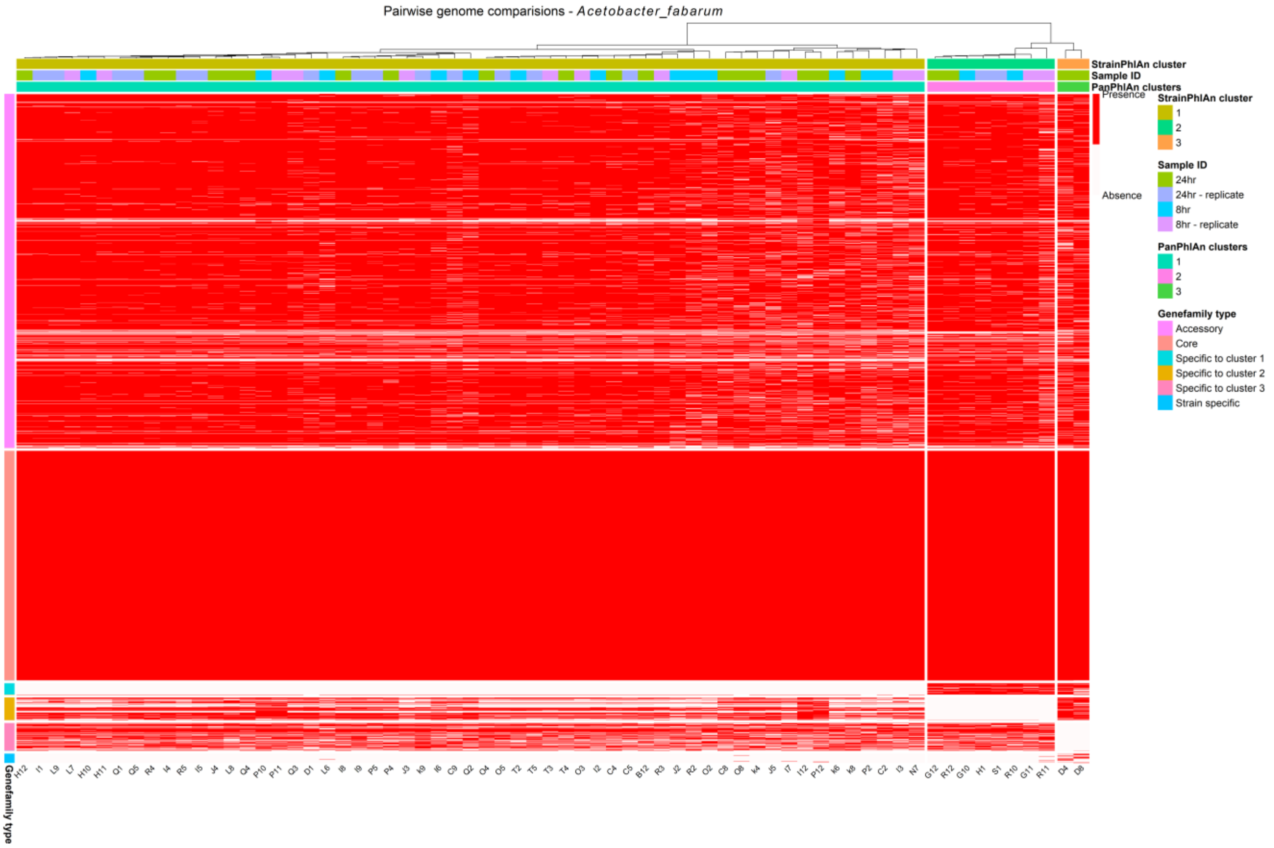

Figure S21. Pangenome of *A. fabarum* strains detected within the milk kefir microbiome, Related to Figure 5. Representative heatmap representing the pangenome of *A. fabarum* strains*;* rows represent PanPhlAn 3 detected strains; columns represent the uniref90 gene families identified per strain. Row side annotations display the grouping of each gene family, e.g., core gene or specific to cluster 1. Column side annotations display the assigned cluster of the detected strain according to the StrainPhlAn 3 and PanPhlAn 3 clustering methodology (see methods) and the time point that the strain was recovered from.

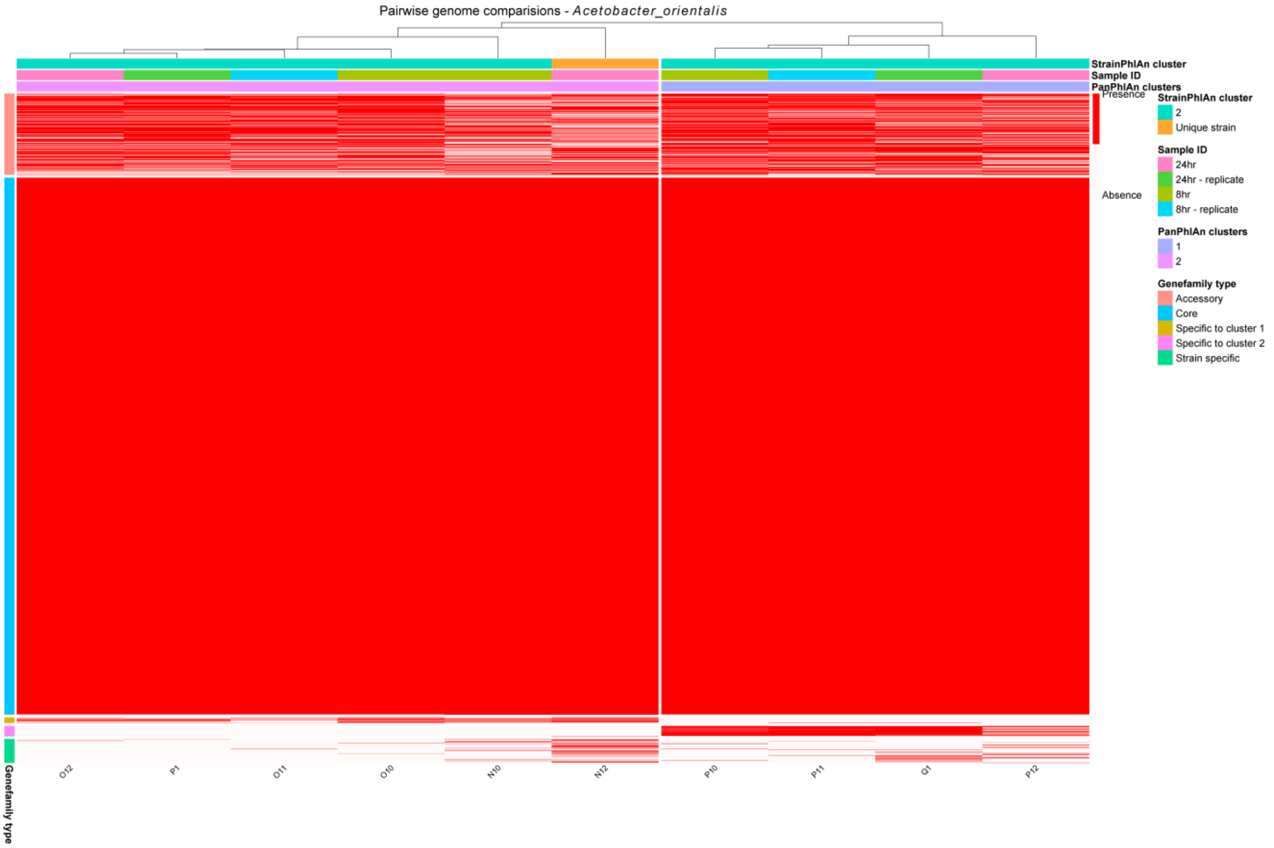

Figure S22. Pangenome of *A. orientalis* strains detected within the milk kefir microbiome, Related to Figure 5. Representative heatmap representing the pangenome of *A. orientalis* strains*;* rows represent PanPhlAn 3 detected strains; columns represent the uniref90 gene families identified per strain. Row side annotations display the grouping of each gene family, e.g., core gene or specific to cluster 1. Column side annotations display the assigned cluster of the detected strain according to the StrainPhlAn 3 and PanPhlAn 3 clustering methodology (see methods) and the time point that the strain was recovered from.

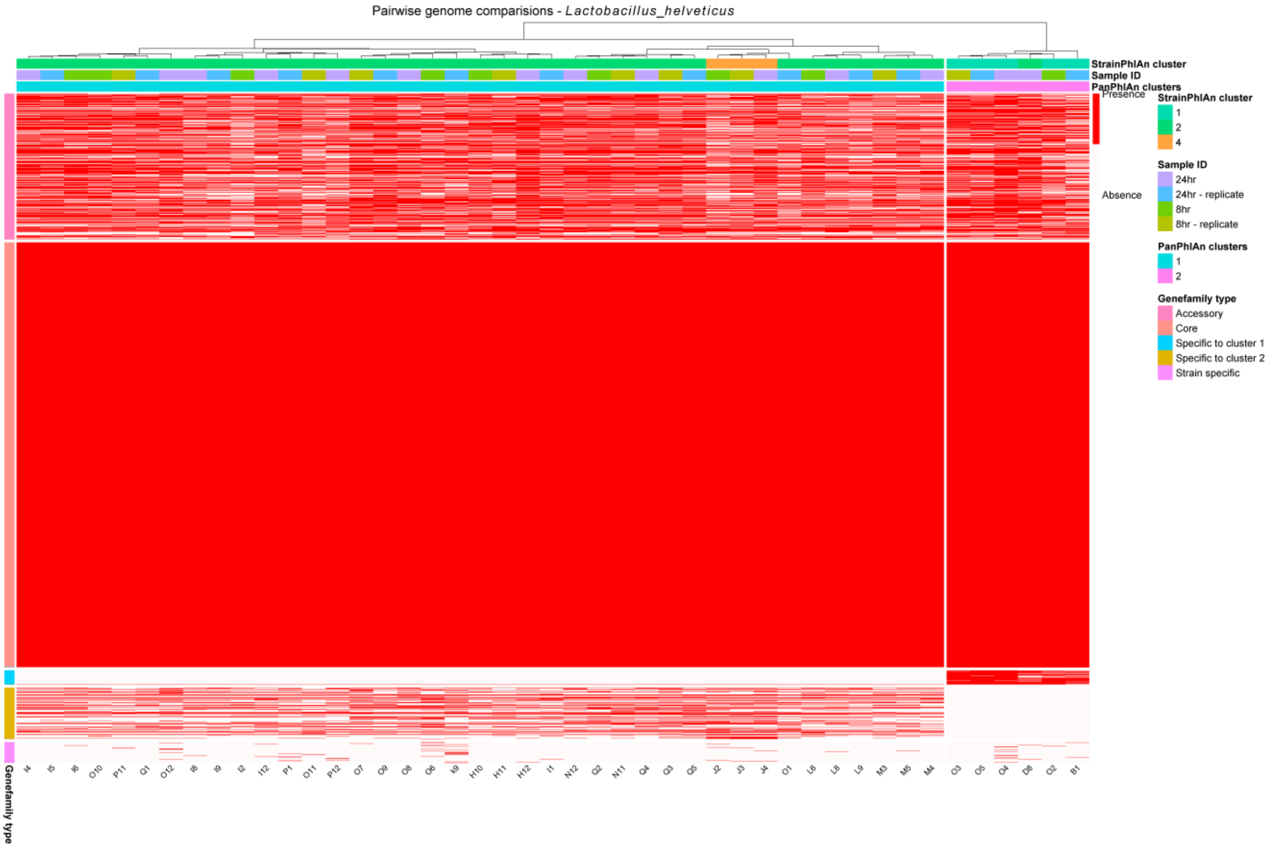

Figure S23. Pangenome of *L. helveticus* strains detected within the milk kefir microbiome, Related to Figure 5. Representative heatmap representing the pangenome of *L. helveticus* strains*;* rows represent PanPhlAn 3 detected strains; columns represent the uniref90 gene families identified per strain. Row side annotations display the grouping of each gene family, e.g., core gene or specific to cluster 1. Column side annotations display the assigned cluster of the detected strain according to the StrainPhlAn 3 and PanPhlAn 3 clustering methodology (see methods) and the time point that the strain was recovered from.

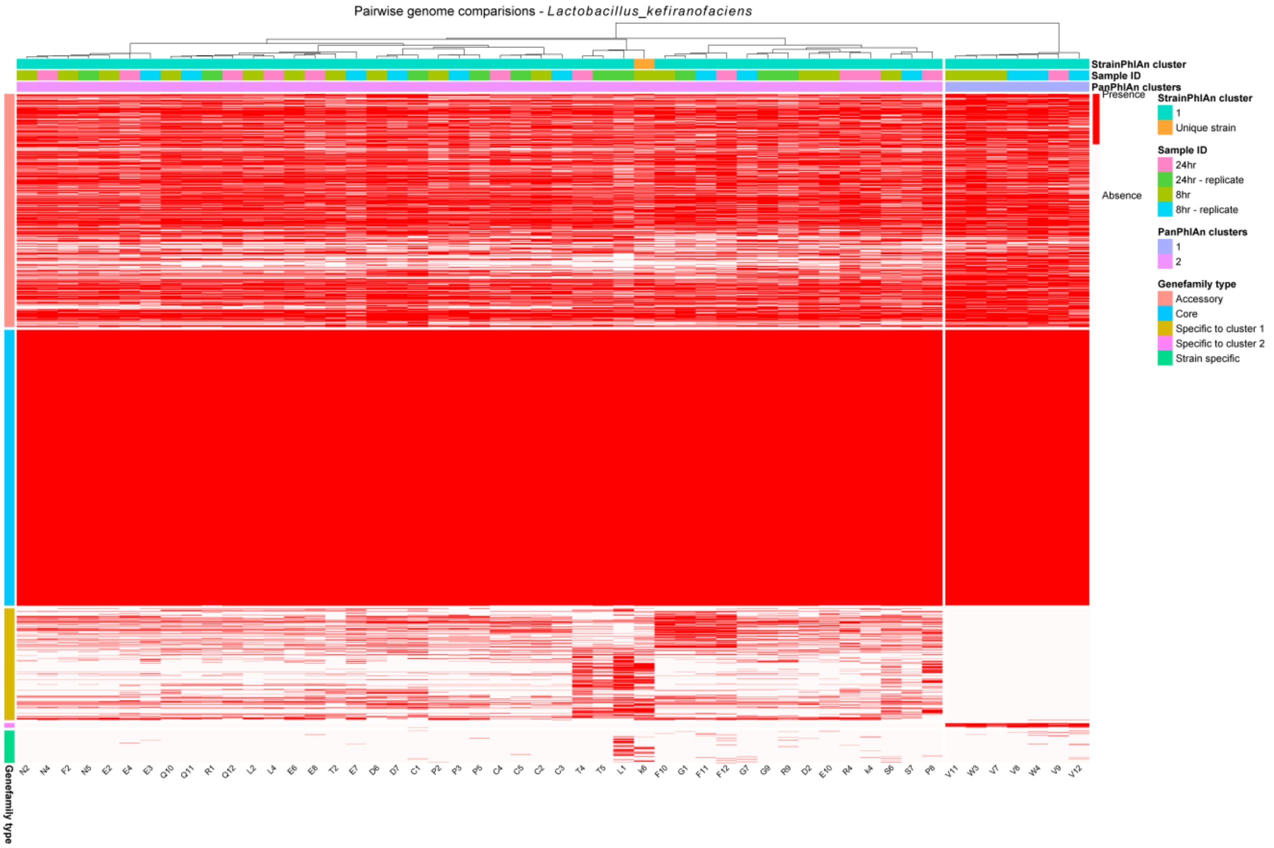

Figure S24. Pangenome of *L. kefiranofaciens* strains detected within the milk kefir microbiome, Related to Figure 5. Representative heatmap representing the pangenome of *L. kefiranofaciens* strains*;* rows represent PanPhlAn 3 detected strains; columns represent the uniref90 gene families identified per strain. Row side annotations display the grouping of each gene family, e.g., core gene or specific to cluster 1. Column side annotations display the assigned cluster of the detected strain according to the StrainPhlAn 3 and PanPhlAn 3 clustering methodology (see methods) and the time point that the strain was recovered from.

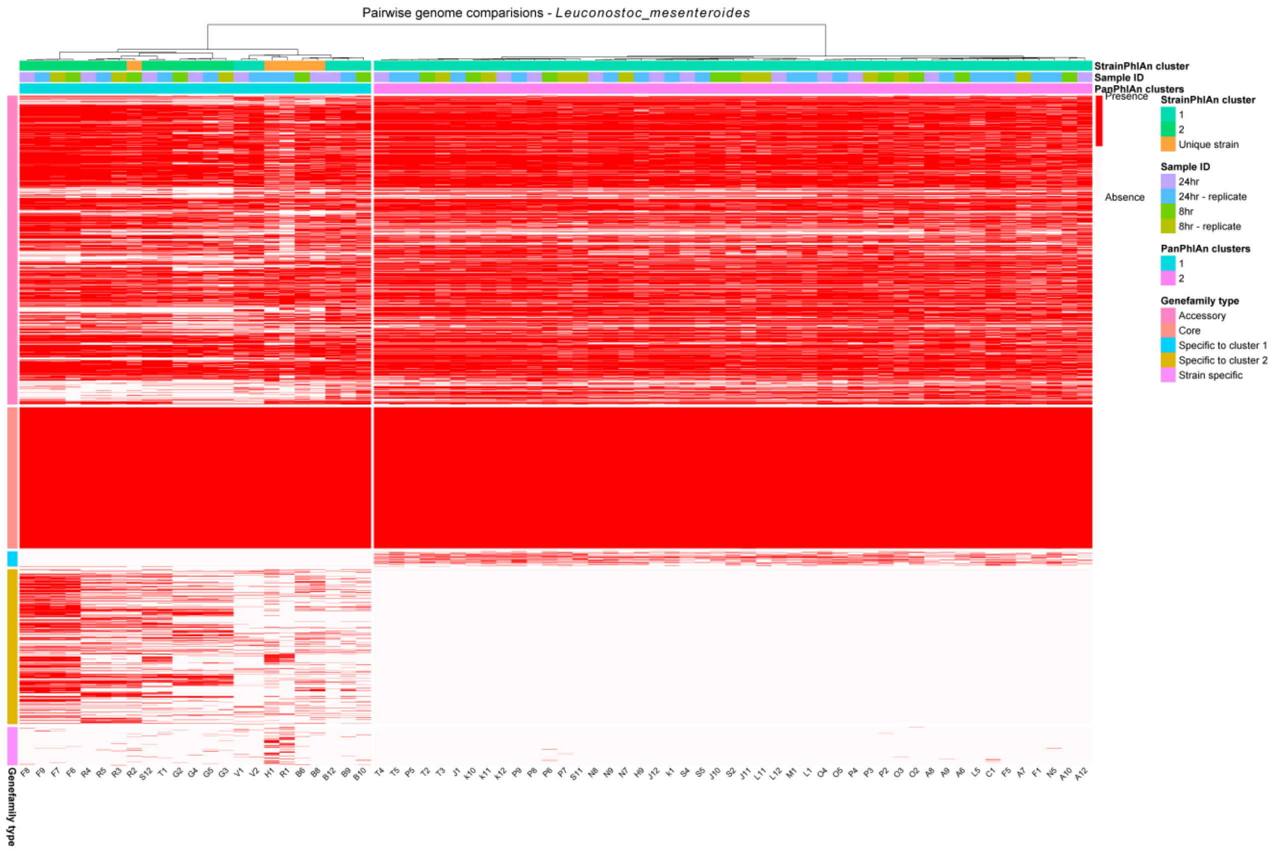

Figure S25. Pangenome of *Leuc. mesenteroides* strains detected within the milk kefir microbiome, Related to Figure 5. Representative heatmap representing the pangenome of *Leuc. mesenteroides* strains; rows represent PanPhlAn 3 detected strains; columns represent the uniref90 gene families identified per strain. Row side annotations display the grouping of each gene family, e.g., core gene or specific to cluster 1. Column side annotations display the assigned cluster of the detected strain according to the StrainPhlAn 3 and PanPhlAn 3 clustering methodology (see methods) and the time point that the strain was recovered from.

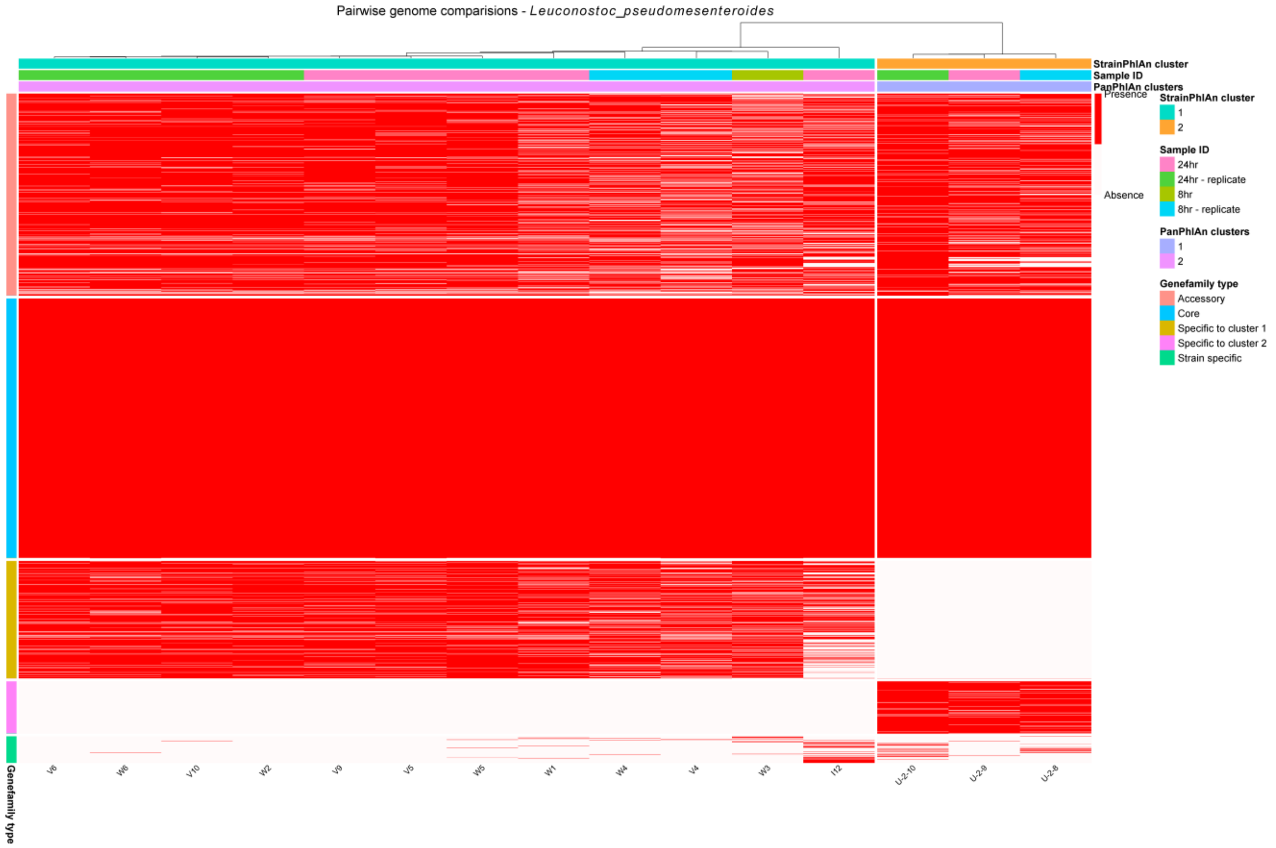

Figure S26. Pangenome of *Leuc. pseudomesenteroides* strains detected within the milk kefir microbiome, Related to Figure 5. Representative heatmap representing the pangenome of *Leuc. pseudomesenteroides* strains; rows represent PanPhlAn 3 detected strains; columns represent the uniref90 gene families identified per strain. Row side annotations display the grouping of each gene family, e.g., core gene or specific to cluster 1. Column side annotations display the assigned cluster of the detected strain according to the StrainPhlAn 3 and PanPhlAn 3 clustering methodology (see methods) and the time point that the strain was recovered from.

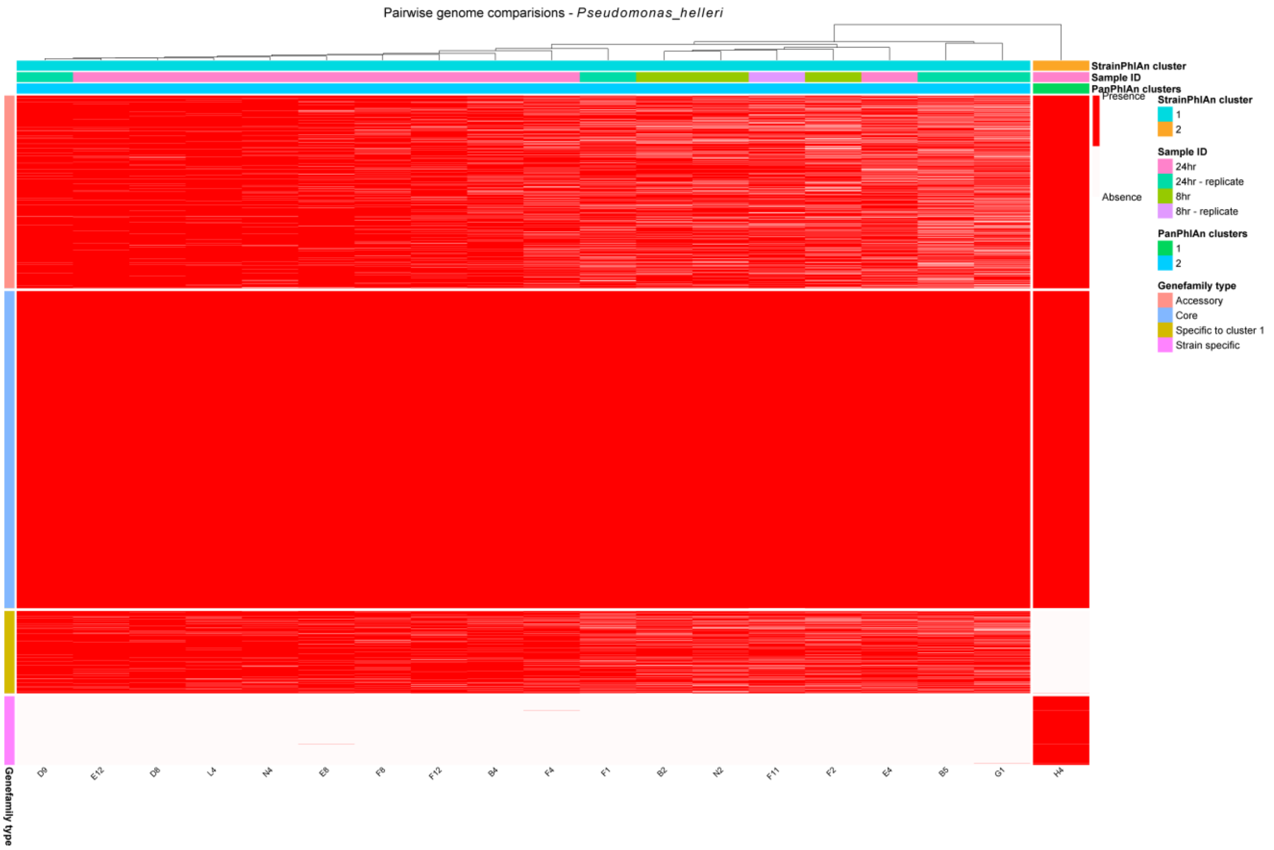

Figure S27. Pangenome of *P. helleri* strains detected within the milk kefir microbiome, Related to Figure 5. Representative heatmap representing the pangenome of *P. helleri* strains; rows represent PanPhlAn 3 detected strains; columns represent the uniref90 gene families identified per strain. Row side annotations display the grouping of each gene family, e.g., core gene or specific to cluster 1. Column side annotations display the assigned cluster of the detected strain according to the StrainPhlAn 3 and PanPhlAn 3 clustering methodology (see methods) and the time point that the strain was recovered from.

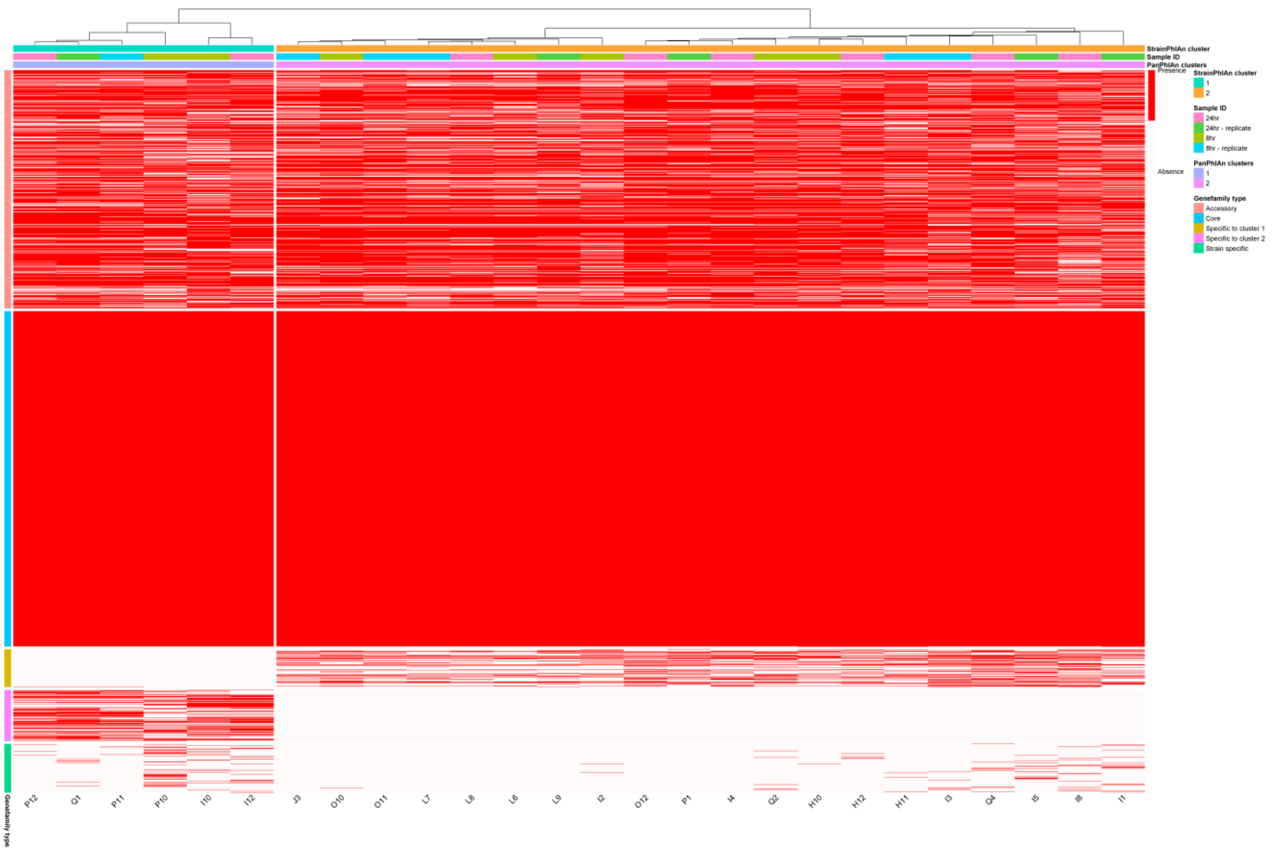

Figure S28. Pangenome of *S. thermophilus strains* strains detected within the milk kefir microbiome, Related to Figure 5. Representative heatmap representing the pangenome of *S. thermophilus* strains; rows represent PanPhlAn 3 detected strains; columns represent the uniref90 gene families identified per strain. Row side annotations display the grouping of each gene family, e.g., core gene or specific to cluster 1. Column side annotations display the assigned cluster of the detected strain according to the StrainPhlAn 3 and PanPhlAn 3 clustering methodology (see methods) and the time point that the strain was recovered from.

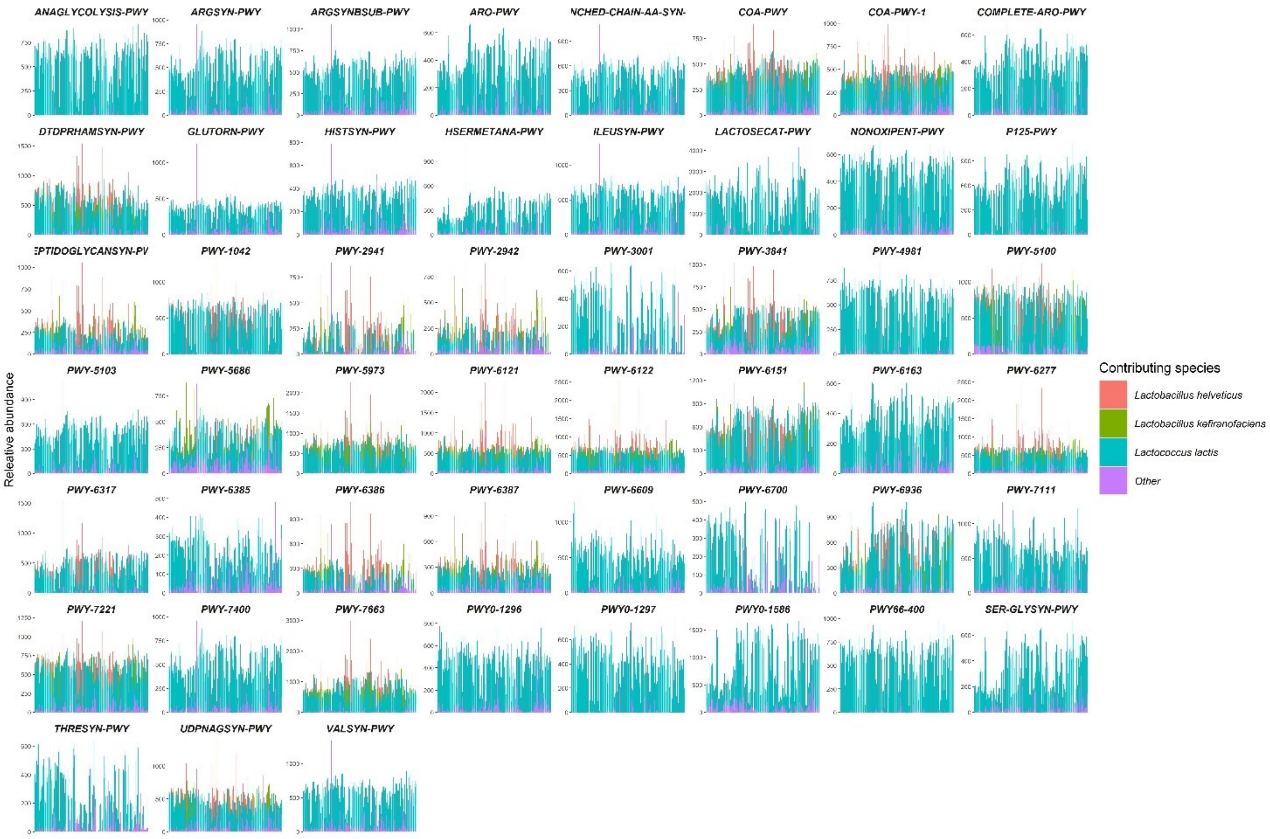

Figure S29. Core metabolic pathway and the associated species in the kefir microbiome, Related to Figure 7. Stacked bar chart of species contributions to the relative abundance of each core metabolic pathway in the kefir microbiome across samples. The contributing species are coloured individually; as *L. helveticus*, *L. kefiranofaciens*, *Lla. lactis and* other
